# How, and For Whom, Does Higher Education Increase Voting?

**DOI:** 10.1007/s11162-022-09717-4

**Published:** 2022-09-14

**Authors:** Caitlin E. Ahearn, Jennie E. Brand, Xiang Zhou

**Affiliations:** 1 University of California—Los Angeles, Los Angeles, CA, USA; 2 Harvard University, Cambridge, MA, USA

**Keywords:** College, Voting, Causal mediation, Heterogeneity

## Abstract

The college-educated are more likely to vote than are those with less education. Prior research suggests that the effect of college attendance on voting operates directly, by increasing an individual’s interest and engagement in politics through social networks or human capital accumulation. College may also increase voting indirectly by leading to degree attainment and increasing socioeconomic status, thus facilitating political participation. However, few studies have empirically tested these direct and indirect pathways or examined how these effects vary across individuals. To bridge this gap, we employ a nonparametric causal mediation analysis to examine the total, direct, and indirect effects of college attendance on voting and how these effects differ across individuals with different propensities of attending college. Using data from the 1979 and 1997 cohorts of National Longitudinal Surveys of Youth, we find large direct effects of college on self-reported voting and comparably smaller indirect effects that operate through degree completion and socioeconomic attainment. We find the largest impact of college on voting for individuals unlikely to attend, a pattern due primarily to heterogeneity in the direct effect of college. Our findings suggest that civic returns to college are not contingent upon degree completion or socioeconomic returns. An exclusive focus on the economic returns to college can mask the broader societal benefits of expanding higher education to disadvantaged youth.

Voting is the primary way citizens participate in democratic politics, by electing representatives and shaping policy outcomes. Yet in each election cycle, millions of eligible voters in the United States choose to stay home, with vast implications for representative democracy. Voter turnout is typically 55–60 percent for presidential elections, 40–45 percent for midterm congressional elections, and generally even lower for local elections (U.S. Bureau of the Census, 2020b). Moreover, voting in the United States, and political participation more broadly, is strongly patterned by educational attainment (Leighley & Nagler, 2013; Putnam, 2001). According to Current Population Survey data, college-educated individuals are 50 percent more likely to vote than those with only a high school diploma (U.S. Bureau of the Census, 2020c). Inequality in voter turnout means that the needs and interests of highly-educated citizens may be overrepresented relative to those of less-educated citizens (Bartels, 2018; Leighley & Nagler, 2013).

Although educational institutions have historically sought to develop civic-minded and engaged citizens (Labaree, 1997), some scholars have questioned the causal relationship between college and political participation. They contend that this association may be spurious due to college-goers being more primed for political engagement than non-college-goers (Highton, 2009; Kam & Palmer, 2008; Tenn, 2007). This body of research, however, has generally ignored the possibility that the effects of college may systematically differ across the population. Another line of research, by contrast, suggests that the impact of college on various civic and socioeconomic outcomes may be strongest for those with lower propensities to attend college—i.e., individuals from more disadvantaged backgrounds and with lower academic ability and achievement (Brand, 2010; Brand & Xie, 2010; Brand et al. 2021; Card, 2001; Heckman et al., 2018; Skinner & Doyle, 2021). If the effect of college on voting follows this pattern of heterogeneity, expanding higher education may lead to greater political engagement among currently underrepresented citizens.

Closely related to heterogeneity in the effects of college are the mechanisms through which those effects operate. Scholars have advanced several theories for how higher education might increase voter turnout. Higher education might directly increase voting through socialization on campus (Bowman, 2011; Chatfield & Henderson, 2016; Glynn et al., 2009), the development of civic literacy (Cassel & Lo, 1997; Galston, 2001; Hillygus, 2005), and politically-engaged social networks (Hansen & Tyner, 2019; Kingston et al., 2003; Rolfe, 2012). Alternatively, a college education may increase voting indirectly, primarily by boosting an individual’s SES, thereby increasing access to occupational networks that foster political engagement (Brady et al., 1995; Cassel & Lo, 1997; Kingston et al., 2003). Scholars have also identified bachelor’s degree completion as a key pathway through which individuals realize the returns to higher education (Hout, 2012). Individuals who attend college may need to complete a degree to realize the civic returns.

Disentangling the contributions of these direct and indirect effects of college on voting provides insight into the relationship between the pecuniary and non-pecuniary benefits of college enrollment. For example, if the voting returns to college depend heavily on post-college socioeconomic attainment, voter turnout should increase most among the subset of college-goers who experience the largest SES returns to higher education. Thus, assessing the degree to which SES mediates the effect of college is essential to understanding not only the extent to which higher education might increase voter turnout but also how it shapes participatory inequality. The proposed direct (Galston, 2001; Hillygus, 2005) and indirect (Brady et al., 1995; Kingston et al., 2003) pathways may operate more strongly among disadvantaged college-goers. First, colleges’ civic missions and liberal education may be particularly consequential for first-generation and lower-income college students, whose social and cultural background may not have as expressly cultivated the commitment to political engagement as that of their more advantaged peers. Higher education may also counter sociopolitical factors and social messaging that suggest to disadvantaged citizens that their votes are inconsequential (Bartels, 2018; Wolak, 2018). Second, if college enhances the SES of individuals from disadvantaged origins to a greater extent than their more advantaged peers (Brand [forthcoming]; Brand & Xie, 2010; Brand et al. 2021; Card, 2001; Heckman et al., 2018), the former group will likewise benefit more from the indirect effects of college.

To date, few studies have jointly considered the direct and indirect pathways through which college affects voting or explored how those pathways vary across the population. Using a novel approach to causal mediation analysis that can accommodate multiple, causally dependent mediators (Zhou & Yamamoto, 2022), this study examines the total, direct, and indirect effects of 4-year college attendance on self-reported voting. We also investigate how these effects vary across individuals with different propensities of attending college. Thus, we show not only the degree to which the civic returns to college attendance depend on degree attainment and socioeconomic returns, but also how this dependence differs between individuals with different characteristics. We use data from the National Longitudinal Survey of Youth 1979 and 1997 cohorts. The former allows individuals two decades to realize the socioeconomic returns to college, thus providing a credible assessment of the mediating role of SES in the effect of college on self-reported voting. The latter offers a comparison of the total, direct, and indirect effects through completion for a more recent cohort.

Our study yields several noteworthy findings. First, the direct effects of college attendance on self-reported voting are larger than the indirect effects through degree completion or SES attainment. Moreover, both the total and direct effects of college attendance are strongest for low-propensity individuals, suggesting a pattern of “negative selection.” The contribution of this study is thus three-fold. First, we contribute to the debate on the civic benefits of higher education by providing strong evidence that 4-year college attendance increases political participation and that the effects are largest among more disadvantaged youth, i.e., those who are least likely to attend college. Second, using a novel approach to causal mediation analysis, we advance understanding of the mechanisms by which college increases political participation. Finally, our results suggest that the civic returns to college are not contingent upon the socioeconomic returns or even degree completion and that an expansion of higher education has the potential to narrow the socioeconomic gap in political participation and influence.

## Background

### The Effect of Education on Voting

Education is one of the most consistent and strongest correlates of voting (Brady et al., 1995; Hauser, 2000; Olsen, 1972; Putnam, 2001). Yet, some have questioned whether the association is causal, or if college simply serves as a proxy for the effects of social background, pre-college cognitive ability, or psychosocial skills (Berinsky & Lenz, 2010; Cassel & Lo, 1997; Dee, 2004; Highton, 2009; Kam & Palmer, 2008; Tenn, 2007). To address the issue of selection bias, scholars have used covariate adjustment, instrumental variable methods, and machine learning approaches. Many studies find positive causal effects of education on political participation (Dee, 2004; Doyle & Skinner, 2017; Mayer, 2011; Milligan et al., 2004; Skinner & Doyle, 2021), while others argue that the relationship is largely spurious (Kam & Palmer, 2008; Quintelier, 2010; Tenn, 2007).

To make sense of these ostensibly conflicting findings, we note that most prior research on the effect of college on political participation has focused on average causal effects, without attending to potential effect heterogeneity. The divergence in findings may be reconciled if we examine heterogeneity in the effects of college on political participation. Studies that report positive effects of college on political participation have often used instrumental variable methods that leverage exogenous variation in compulsory schooling laws, child labor laws, or the availability of community colleges (e.g., Dee, 2004; Doyle & Skinner, 2017; Heckman et al., 2018; Milligan et al., 2004). If there is heterogeneity in the effect of college on voting, then these results apply to students induced into college by the instrument, who are typically relatively disadvantaged and less likely to attend college than the average college-goer. For example, Doyle & Skinner (2017) find that IV estimates of college effects exceed standard OLS estimates for self-reported voting and conclude that civic benefits to higher education appear to accrue largely for individuals on the margins of attendance. Moreover, Skinner & Doyle (2021) find somewhat larger effects of college enrollment on voter registration among those with a lower propensity to enroll.

Additionally, a body of higher education research has found that the effects of 4-year college on socioeconomic and civic outcomes differ across the population. In particular, the effects of college appear to vary by selection into college, such that individuals with a lower likelihood of attending and completing college experience different returns from college than those with a higher likelihood (Brand [forthcoming]; Brand, 2010; Brand & Xie, 2010; Card, 2001; Cheng et al., 2021; Heckman et al., 2018; Zhou & Xie, 2020). Some research suggests that the effect of college on civic engagement and self-reported voting is largest for individuals who are unlikely to attend and complete college (Brand, 2010; Heckman et al., 2018).

In this study, we contribute to the growing literature on heterogeneous college effects by examining how the effect of college on political participation varies by selection into college. We attend to differential selection bias across subpopulations of interest and assess the sensitivity of our results to different forms of unobserved confounding.

### Mechanisms Linking College and Voting

Examining potential mechanisms is critical to understanding the relationship between higher education and voting and why particular subgroups may gain more from college than others. Scholars have suggested both direct and indirect pathways through which four-year college increases voting: directly through socialization and the development of civic literacy and indirectly through socioeconomic attainment and family formation. As an important place of socialization, college can provide access to diverse social experiences, which may contribute to developing a social identity geared toward civic and political participation (Stevens et al., 2008). Exposure to racial and ethnic diversity fosters interactions with diverse peers, which can increase civic and political engagement (Bowman, 2011). Relatedly, campus social norms that encourage political participation are associated with higher rates of voting (Glynn et al., 2009). The social theory of political participation suggests that more educated citizens are more likely to vote due to more cohesive social networks and stronger social norms around voting (Hansen & Tyner, 2019; Rolfe, 2012). Social networks developed because of higher education can have lasting effects on political participation into adulthood. Alternatively, negative social experiences on campus, such as racially motivated microaggressions, may spur students to civic action (Baker & Blissett, 2018; Skinner & Doyle, 2021). The social aspect of college attendance may thus directly encourage individuals toward political participation.

Higher education can also increase civic literacy and subsequent political participation (Cassel & Lo, 1997; Chatfield & Henderson, 2016; Galston, 2001; Hauser, 2000; Hillygus, 2005; Milligan et al., 2004). Certain types of college learning experiences may have greater impacts on political literacy than others. For instance, civic and political learning is more common in majors that emphasize the political system, social institutions, and inequality (Hillygus, 2005; Quintelier, 2010) or in liberal arts programs that emphasize general learning (Arum & Roksa, 2010). Given that socialization and civic literacy are part of the college experience, and social networks developed during college often extend into adulthood, we characterize these processes as constituting the *direct* effect of college on voting.

The effect of college attendance on voting may also operate through a mediator that is both an outcome of college attendance and a cause of increased political participation. One potential mediator is the attainment of a bachelor’s degree. Previous research has found that a bachelor’s degree operates as a gatekeeper to socioeconomic returns to college attendance (Hout, 2012), but whether civic returns similarly require degree completion remains uncertain. We thus distinguish the direct effect of college attendance from the indirect effect via college completion to assess whether college boosts voting regardless of or because of bachelor’s degree attainment.

It is well established that a college education positively affects socioeconomic status (SES), be it measured by individual earnings, family income, employment, or occupational status (Hout, 2012; Sewell & Hauser, 1975). Previous studies suggest that both income (Freeman, 2003; Leighley & Nagler, 2013; but see Brady et al. (1995) and Wolfinger & Rosenstone, (1980) for some counter-evidence) and occupational position (Freeman, 2003) are positively associated with the likelihood of voting. Brady et al. (1995) argue that occupational experiences help workers develop civic skills. The occupations of college-educated workers tend to cultivate networking, writing, and public speaking skills, which consequently increase their political engagement. College graduates may also experience greater encouragement to participate in political activities, possibly because they are perceived as having greater access to socioeconomic resources or more influential networks (Hauser, 2000). According to this argument, SES “is critical in shaping interests, outlooks, and social behavior,” rendering education “socially consequential only indirectly through its impact on economic status” (Kingston et al., 2003, p. 57).

In these cases, greater socioeconomic attainment is understood to increase access to and social encouragement for political activities, including voting. A corollary of this process is that lower-SES citizens face socioeconomically-driven obstacles that reduce their likelihood of voting. Such obstacles might be a lack of reliable transportation to the polls (U.S. Bureau of the Census, 2012), frequent moving that prevents registration (Phinney, 2013; Wolfinger & Rosenstone, 1980), or other adversities that demand their time and energy. Thus, college may increase voting indirectly through SES not only by increasing the proclivity to vote but also by reducing socioeconomic barriers to political engagement.

Education also has important effects on family outcomes, including increased likelihood of marriage (Schneider, 2011), reduced likelihood of single parenthood (Lundberg et al., 2016), and increased marital stability (Amato, 2010). Married individuals consistently exhibit higher rates of political participation than others (Leighley & Nagler, 2013). College may thus also boost voting through its impact on family formation and stability. Moreover, because family formation and stability itself facilitate socioeconomic attainment (e.g., Killewald & Gough, 2013; Smock et al., 1999), the mediating effect of family may further operate through SES, constituting a causal chain characterized by education → family → SES → voting.

### Heterogeneity in the Mechanisms Linking College and Voting

As with the total effect of college on voting, there is little reason to assume that the mediating effects are constant across the population. While no prior research (to our knowledge) has considered potential heterogeneity in the mediating pathways between college and voting, prior work on heterogeneous economic returns to college offers some directions to consider. In particular, the effects of four-year college attendance and completion on earnings appear to be high for individuals who are less likely to attend and complete college (Brand & Xie, 2010; Card, 2001; Cheng et al., 2021). Relatedly, recent research suggests that college circumvents socioeconomic disadvantage for those on the margin of school continuation (Brand [forthcoming]; Brand et al. 2021; Heckman et al., 2018). From this perspective, we would expect the indirect effect of college via SES to be greater for individuals who are unlikely to attend college. The direct effects of college on voting may similarly vary. Students from more disadvantaged backgrounds or from racially minoritized groups, who are more commonly on the margin of four-year college attendance, may have social encounters on college campuses that encourage social activism (Baker and Blissett 2018; Jack, 2019). Likewise, more disadvantaged college-goers may face lower pre-college social pressure to vote, and thus may face greater increases in positive social pressure from college attendance.

Scholars have shown that the effects of college attendance on marriage and marital stability vary with the propensity to complete college. In this case, however, the largest effects accrue to those who are most likely to complete college (Musick et al., 2012). On the other hand, the effect of college on reducing fertility is larger among more disadvantaged college-goers (Brand & Davis, 2011). We thus remain agnostic with respect to how the mediating effect of family characteristics varies across population subgroups. We nevertheless include variables related to family formation and marital stability in our models given the important effects of college on family and of family on political participation.

Despite the well-documented association between education and political participation and theories on different mechanisms leading to increased voting, the mediating pathways between college and voting remain poorly understood. Prior studies have not explored different pathways using a causal mediation framework. In addition, no previous research has examined how college’s direct and indirect effects on political participation vary across the population. If college increases voting more strongly for disadvantaged youth, as recent research suggests, studies should focus on mechanisms explaining this important source of heterogeneity. In this paper, we investigate not only the extent to which degree attainment, family characteristics, and socioeconomic status mediate the effect of college on self-reported voting but also how the direct and indirect effects vary by the propensity of attending college.

## Voter Reporting and Representation Bias in Survey Data

Voting rates in survey data tend to be considerably higher than actual turnout rates (Berent et al., 2016; Debell et al., 2020). Scholars have argued that two processes bias survey voting rates: overrepresentation of voters among respondents and vote overreporting among non-voters (Ansolabehere & Hersh, 2012; Enamorado & Imai, 2019; Goldberg & Sciarini, 2019; Selb & Munzert, 2013). Some validation studies, which use official data to assess the accuracy of self-reported voting behavior, have identified high rates of voter overreporting in surveys and have shown that overreporting tends to increase with education (Ansolabehere & Hersh, 2012; Enamorado & Imai, 2019). This suggests that analyses of survey data, such as those in our study, may overstate the effect of education on voting.

On the other hand, the source of the survey turnout gap in this study, and thus the implications for our results, remains unclear. First, validation studies in the US have generally relied on political surveys, such as the American National Election Study (ANES), which draw more politically motivated respondents than a general survey like the NLSY. A less political sample may feel less pressure to misreport their voting behavior, resulting in a smaller turnout gap (Debell et al., 2020). Second, validated data of US voters are imperfect. A recent report by the Pew Research Center (2018) shows that commercial voting data sets tend to be incomplete when examined individually. Even when combined, these data sets are more likely to miss disengaged, young, Hispanic, and low-income individuals (Pew 2018). In an analysis of voter validation methods, Berent et al. (2016) similarly find that “the process of matching government records often failed to locate records of respondents who were truly registered and had voted” (p 615). They argue that matching survey respondents to government records underestimates the proportion of Americans registered to vote. Among respondents whose self-reports can be validated against government records, the accuracy of self-reports is extremely high. Their findings imply that while validated turnout estimates appear to be more accurate than self-reports because they produce lower turnout estimates, “the apparent accuracy is most likely an illusion” (Berent et al., 2016, p. 599).

Moreover, and perhaps more importantly, our primary aim in this study is to assess heterogeneity in the effect of college on voting by the propensity to enroll in college. We find that the effect of college is larger for more marginal college-goers, who tend to be more disadvantaged and lower-achieving. Bias-induced invalidation of these effects would require either more voting overreporting or greater overrepresentation of voters among lower propensity college-goers than their higher propensity peers. To our knowledge, there is no evidence from existing literature that either of these processes is at play.

## Data and Measures

We use data from the 1979 and 1997 National Longitudinal Surveys of Youth (NLSY) cohorts, which follow respondents from adolescence through adulthood. Both surveys interviewed respondents annually or biennially, collecting data on sociodemographic background, academic achievement, attitudes, educational attainment, and family and labor market outcomes during adulthood. Our main analyses use the 1979–2006 waves of the NLSY79 cohort, which began with adolescents aged 14–22 in 1979. We restrict our NLSY79 sample to 14–17-year-old respondents at the baseline survey in 1979 (*n* = 5582) who had completed at least the 12th grade (*n* = 4548). We set these sample restrictions to ensure that all variables used to predict college are measured before college, particularly academic ability, and to compare college-goers to those who completed only a high school education. We also focus on the region of common support in terms of the estimated propensity scores, as described below (*n* = 4085). We further restrict the sample to respondents who answered the question about voting in the 2006 general election (*n* = 2961). This reduction in sample size largely reflects attrition over 27 years of follow-up.

In 2006, the NLSY79 asked respondents whether they voted in the 2006 general election. NLSY political questions are modeled from questions from the American National Election Studies (ANES). Self-reported voting is based on a question that asks respondents which statement best describes them: (1) I am sure I voted; (2) I usually vote, but didn’t in 2006; (3) I thought about voting in 2006, but didn’t; and (4) I did not vote in the November 2006 election. Some respondents refused to answer or reported that they didn’t know. We code those who stated that they are sure they voted as voting and all other non-missing categories as did not vote.^1^ Among NLSY79 respondents who have at least a high school diploma, 69 percent report voting.

As noted, people who do not vote are disproportionately unlikely to participate in surveys. To assess the robustness of our results to potential bias due to the overrepresentation of voters among those who responded to the voting question in NLSY, we have conducted a sensitivity analysis where individuals who were not interviewed for or did not answer the voting question were coded as non-voters. The findings from this analysis are discussed briefly in the Results section and in greater detail in Online Appendix D.^2^

We define the treatment variable as whether a respondent attended a 4-year college by age 20.^3^ Pre-college covariates include individual, school, and family characteristics that are known to predict college attendance. Our sociodemographic characteristics include race, gender, family socioeconomic status, family structure, religion, and region. We also use measures of ability [i.e., the 1980 Armed Services Vocational Aptitude Battery, adjusted for age and standardized following Brand and Xie (2010)], psychosocial characteristics, college preparation and expectations, delinquent activity, high school characteristics, and family formation at age 18.

We examine three pathways through which college attendance may increase voting: bachelor’s degree completion by age 25, family formation and stability during 1990–1998, and socioeconomic attainment during 1998–2006. By temporally ordering these mediators, we disentangle the unique causal paths via college completion, family characteristics, and socioeconomic attainment successively. We use three variables to gauge family formation and stability: the proportion of time respondents were married, the proportion of time they were unmarried parents, and the number of family transitions between 1990 and 1998. To assess socioeconomic attainment, we use a battery of variables reflecting employment and wages, job stability, poverty and social assistance, and residential turnover and homeownership between 1998 and 2006. These include the proportion of time out of the labor force, average wages, number of jobs held, job tenure at age 40, the proportion of time in poverty, cumulative welfare received, number of residential moves between 2000 and 2006, and homeownership status in 2004.

We present descriptive statistics of the political participation outcome, all pre-college covariates, and all family and socioeconomic mediators in Table 1. Our descriptive statistics of pre-college covariates are consistent with well-documented socioeconomic and achievement differences between college- and non-college-goers. Except for a few covariates, these differences are statistically significant at the *p* < 0.05 level. Descriptive statistics of the post-treatment mediators also align with expected differences by college attendance. College-goers have more stable family lives and higher socioeconomic status. As expected, college-goers are more likely to report voting in the 2006 general election than individuals who did not attend college. These differences are also statistically significant at the *p* < 0.05 level.

The mediating effects of family and socioeconomic attainment are realized many years after college attendance. Using the NLSY79 allows sufficient time for these life events to unfold and subsequently influence voting during middle adulthood. However, the experiences of this older cohort, who attended college in the 1980s, may be less applicable to current college students. For this reason, we supplement our main findings with analyses from the 1997–2010 waves of the NLSY97 cohort, which began with adolescents aged 12–17 in 1997. Our outcome is whether respondents voted in the 2010 national election, the only post-college voting item asked of the full NLSY97 sample. Among NLSY97 respondents who have at least a high school diploma, 40 percent report voting in the 2010 election. We use the same approach to construct this measure as for the NLSY79 data. Due to the temporal proximity of the 2010 voting outcome to this cohort’s college completion, we cannot examine the mediating effects of family characteristics and socioeconomic attainment using the NLSY97. Thus, our supplemental analyses focus on estimating heterogeneity in the total effect of college attendance and the mediating effect of college completion on voting. Pre-college covariates used in these analyses, and differences in those covariates by college attendance status, are like those for the NLSY79 cohort. We report descriptive statistics for the NLSY97 variables in Online Appendix A.

## Analytic Strategy

### Estimating Path-Specific Causal Effects

In this study, we aim to disentangle the specific pathways through which college affects political participation. This goal poses a methodological challenge because existing methods for causal mediation analysis have largely focused on the role of a single mediator or of a set of mediators considered as a whole (Baron & Kenny, 1986; Imai et al., 2010; Vander-Weele, 2015). In the presence of multiple mediators, it is often assumed that these mediators are causally independent, i.e., they do not affect each other. This assumption, however, is strong, untestable, and unrealistic in our application. It is well documented that college completion, family formation, and marital stability influence individuals’ socioeconomic status (Hout, 2012; Killewald & Gough, 2013; Smock et al., 1999).

**Fig. 1:**
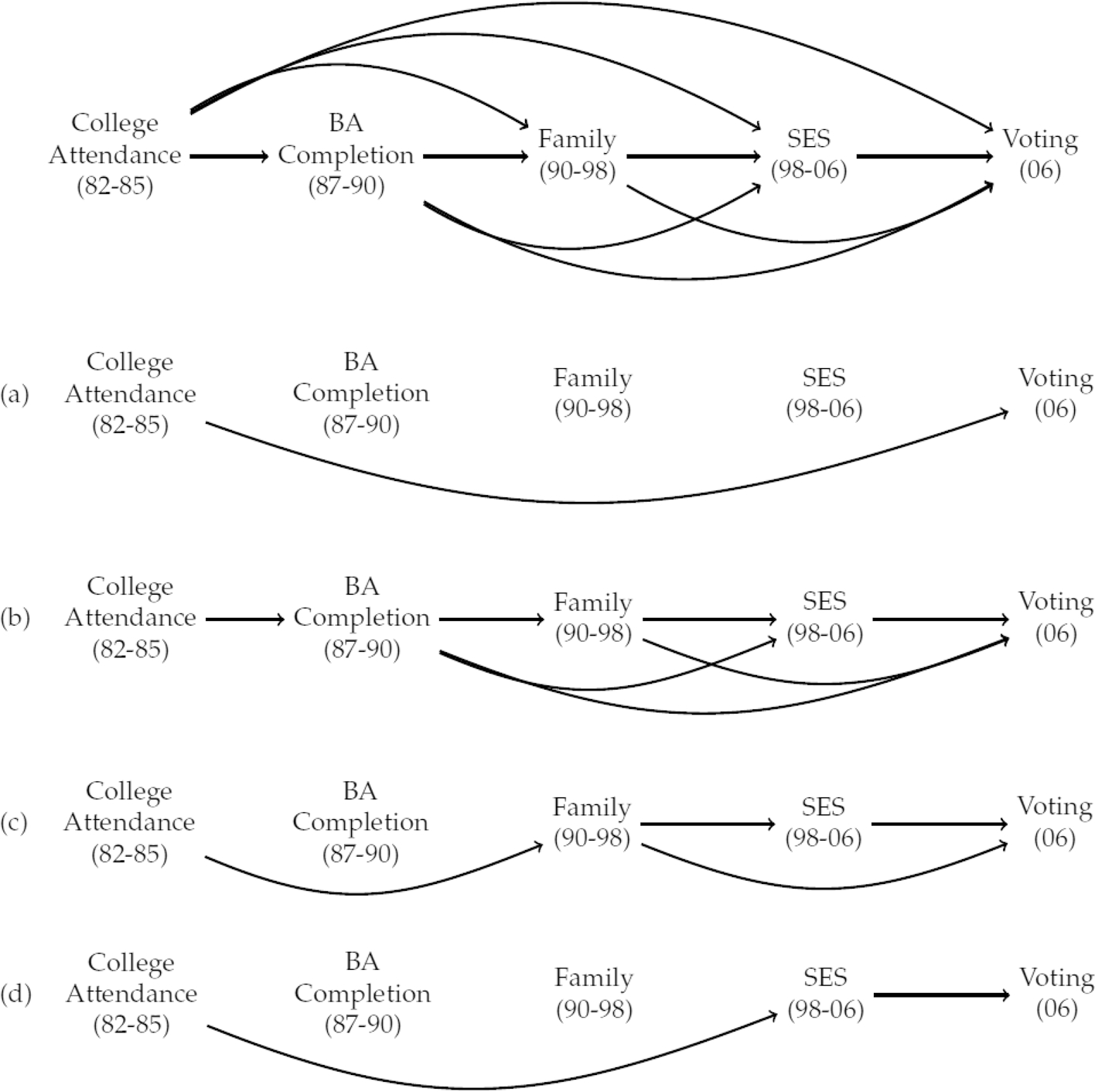
Causal pathways through which college may affect voting. Numbers in parentheses indicate the years in which the corresponding variables are measured

To accommodate the reality that our three mediators are causally dependent, we analyze the causal diagram shown in the upper panel of Fig. 1. This diagram consists of five sets of temporally ordered and causally dependent variables from the NLSY79: (1) 4-year college attendance by age 20; (2) 4-year college completion by age 25; (3) variables reflecting family characteristics from about age 25 to the mid-30 s (i.e., in 1990–1998); (4) variables reflecting SES from the mid-30s to early 40s (i.e., in 1998–2006); and (5) voting participation in the early 40s (i.e., in 2006). In this diagram, the baseline covariates that we use to adjust for confounding are omitted. The lower four panels of Fig. 1 represent four possible mechanisms through which college attendance increases voting: (a) college attendance may increase voting directly; (b) college attendance may influence college completion, which in turn affects voting either directly or via family characteristics and/or SES; (c) regardless of college completion status, college attendance may influence family formation and stability, which in turn affects voting either directly or through increased SES; (d) regardless of college completion status and family characteristics, college attendance may increase SES, which in turn leads to higher levels of voting.

Following Pearl (2009), we interpret a causal diagram as a nonparametric structural equation model with independent errors. This model assumes no unobserved confounding exists for any treatment-mediator, treatment-outcome, mediator-mediator, or mediator-outcome relationships. In our context, it implies that conditional on the precollege covariates described in the previous section, (a) no unobserved variables affect both college attendance and any of the college completion, family, SES, and voting outcomes; (b) no unobserved variables affect both college completion and any of the family, SES, and voting outcomes; (c) no unobserved variables affect both family characteristics and any of the SES and voting outcomes; and (d) no unobserved variables affect both SES and voting. Recognizing that these assumptions are strong and untestable, we conduct a series of sensitivity analyses that investigates the direction and magnitude of potential bias when some of these assumptions are violated. These analyses are described briefly in the Results section and in more detail in Online Appendix D.

Under the above assumptions, the path-specific effects corresponding to mechanisms (a), (b), (c), and (d) in Fig. 1 are nonparametrically identified (Avin et al., 2005; Vander-Weele, 2015; Zhou, 2022), i.e., expressed in terms of observed data without any functional form assumption. Specifically, if we let *A* denote treatment (i.e., college attendance), Y the outcome of interest (i.e., voted in 2006), X the vector of pretreatment covariates, C the college completion mediator, L the set of mediators reflecting family formation and stability, and M the set of mediators reflecting SES, the average total effect (ATE) of college on voting can be decomposed into four components^4^:

(1) $$ATE=\tau_{A\to Y}+\tau_{A\to CY}+\tau_{A\to LY}+\tau_{A\to M\to Y},$$

where the quantities $\tau_{A\to Y},\tau_{A\to CY},\tau_{A\to LY}$ and $\tau_{A\to M\to Y}$ reflect panels (a), (b), (c), and (d) in Fig. 1, respectively. Their detailed expressions and further explanation are given in Online Appendix B.

To estimate these path-specific effects, we use an imputation estimator proposed in Zhou & Yamamoto (2022), which involves fitting four outcome models for voting participation. The first is conditional on college attendance and the baseline covariates only (i.e., a model for E[Y∣X,A])). The second is conditional on college attendance, the baseline covariates, and college completion (i.e., a model for E[Y∣X,A,C]). The third is conditional on the college attendance, baseline covariates, college completion, and the mediators reflecting family formation and stability in 1990–98 (i.e., a model for E[Y∣X,A,C,L]). The fourth is conditional on college attendance, the baseline covariates, college completion, the mediators reflecting family formation and stability in 1990–98, and the mediators reflecting SES in 1998–2006 (i.e., a model for E[Y∣X,A,C,L,M]. After fitting these outcome models, we fit three additional models for the conditional expectations: E[E[X,A=1,C]∣X,A], E[E[X,A=1,C,L]∣X,A] and E[E[X,A=1,C,L,M]∣X,A], which are then used to evaluate the path-specific effects in Eq. (1).

We use boosted regression trees (Friedman, 2001), a flexible machine learning method with strong predictive accuracy, to fit the above models. We use the R package *gbm* (Ridgeway, 2019) to construct the regression trees, allowing for two-way interactions and using a learning rate of 0.02. The numbers of trees used for these outcome models are selected via five-fold cross-validation. Standard errors and confidence intervals for the path-specific effects are estimated via the nonparametric bootstrap.

### Evaluating Treatment Effect Heterogeneity

In addition to assessing the average total effect of college and its direct and indirect components, we also investigate how these effects vary across individuals. Following previous research on heterogeneous effects of college on socioeconomic and civic outcomes (e.g., Brand, 2010; Brand & Xie, 2010; Cheng et al., 2021), we use the propensity score of attending college as a summary index of pre-college advantage, i.e., the conditional probability of attending college given the precollege covariates described in the previous section. As a robustness check, we also examined heterogeneity by two alternative indicators of socioeconomic and academic advantage: parental income and measured ability.

We fit the propensity score models for college attendance using an iterative procedure outlined by Imbens & Rubin (2015), which leads to a fairly flexible specification with good balancing properties. After beginning with a set of key baseline covariates, we considered additional possible covariates in turn. We identified covariates, including higher-order and interaction terms, that produce a likelihood ratio statistic that exceeds a pre-set constant when added to the model. This procedure involved roughly 5800 regressions and resulted in a model with 23 linear terms, one higher-order term, and 19 interaction terms. The final set of covariates identified through this process is described above. We further eliminated units with no common support, i.e., treated cases with values higher than the highest propensity score among the controls (p(X)=0.921;n=13) and untreated cases with values lower than the lowest propensity score among the treated (p(X)=0.002;n=411).

To assess the heterogeneity of the direct and path-specific effects described previously, we extract the “individual-specific” treatment effect estimates inside the expectation operators derived from the expressions of the components of Eq. 1 (see Online Appendix B). Then, for each of these individual-specific estimates of $\tau_{A\to Y},\tau_{A\to CY},\tau_{A\to LY}$, and $\tau_{A\to M\to Y}$, we fit a regression model for their conditional mean given the estimated propensity score (in auxiliary analyses, parental income and cognitive ability). To allow for sufficient nonlinearity, we use a smoothing spline with three degrees of freedom (Hastie, 2017). Standard errors and confidence intervals for these conditional means are estimated via the nonparametric bootstrap.

## Results

### Total and Path-Specific Effects of College Attendance on Voting

**Fig. 2:**
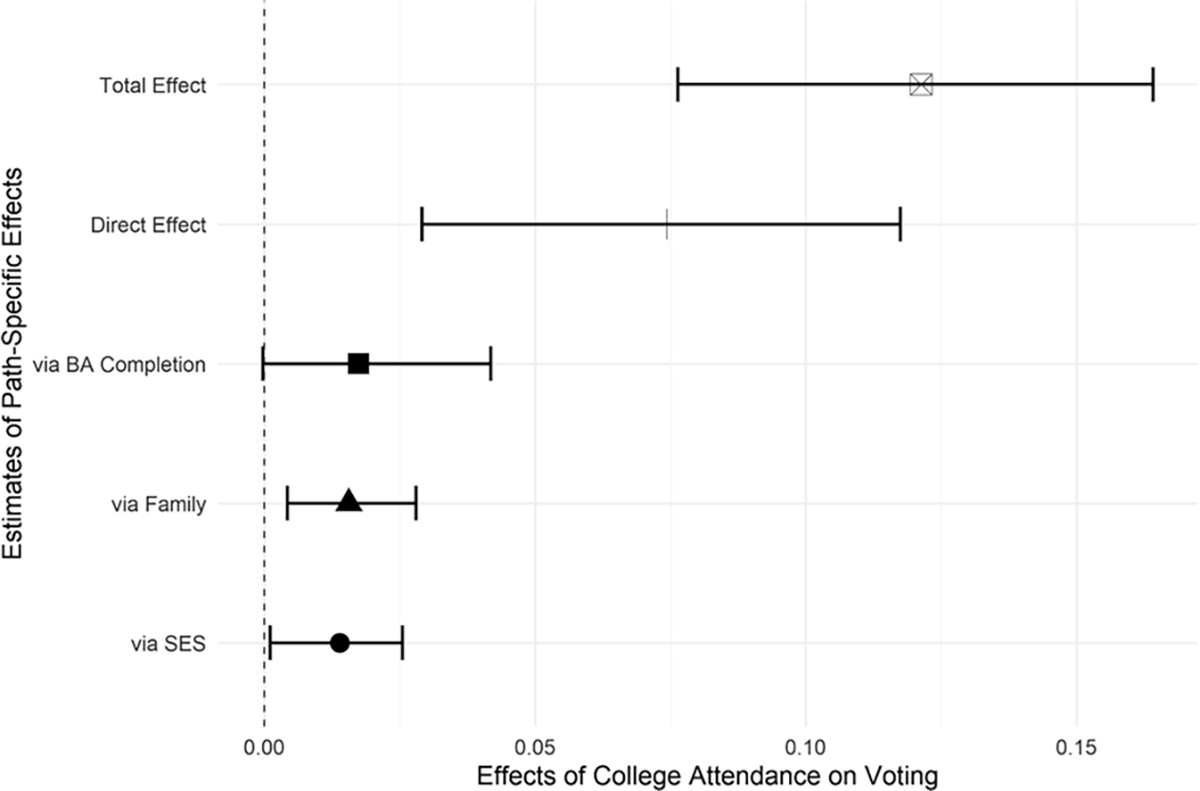
Estimated path-specific effects of college attendance on voting with 95% bootstrap confidence intervals (1000 iterations), NLSY79

We begin by examining the total and path-specific effects of 4-year college attendance on voting for the overall sample. Results are presented in Fig. 2. The estimated effects in these figures reflect the percentage-point increase in the rate of self-reported voting associated with attending college. Figure 2 indicates that, overall, college attendance increases self-reported voting rates by about 12 percentage points. The estimated direct effect of college attendance is slightly less than two-thirds of the total effect (7.5 percentage points), and the estimated combined mediating effect of bachelor’s degree completion, family formation, and socioeconomic attainment accounts for the remaining 4.5 percentage points.^5^ This suggests that the effect of college attendance on voting operates primarily directly, whereas the mediating effects of degree completion, family, and SES are comparatively weaker (though still significant).

### Heterogeneous Effects of College Attendance on Voting

**Fig. 3:**
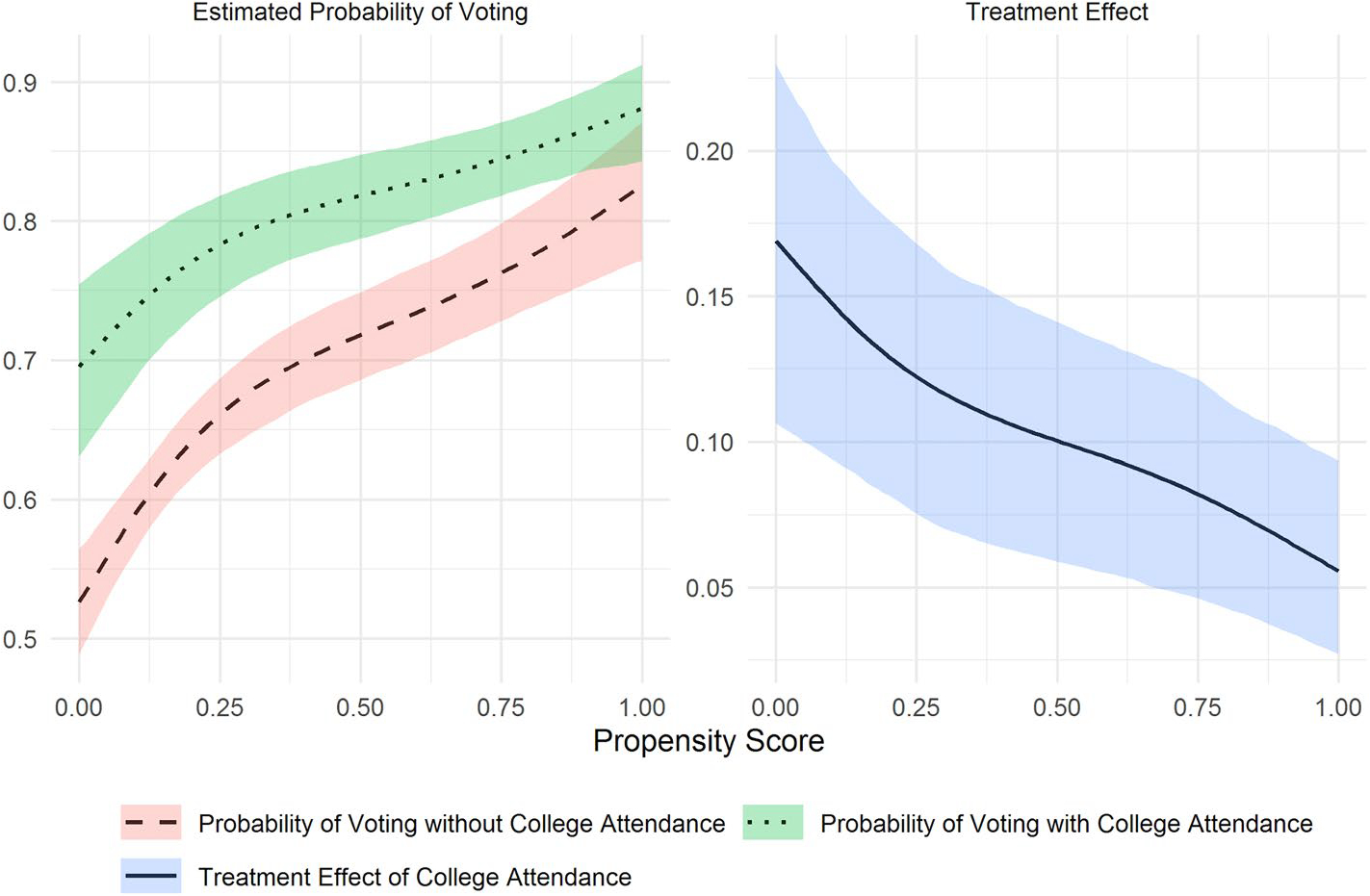
Heterogeneous total effects of college attendance on voting by propensity scores with 95% bootstrap confidence bands (1000 iterations), NLSY79

**Fig. 4:**
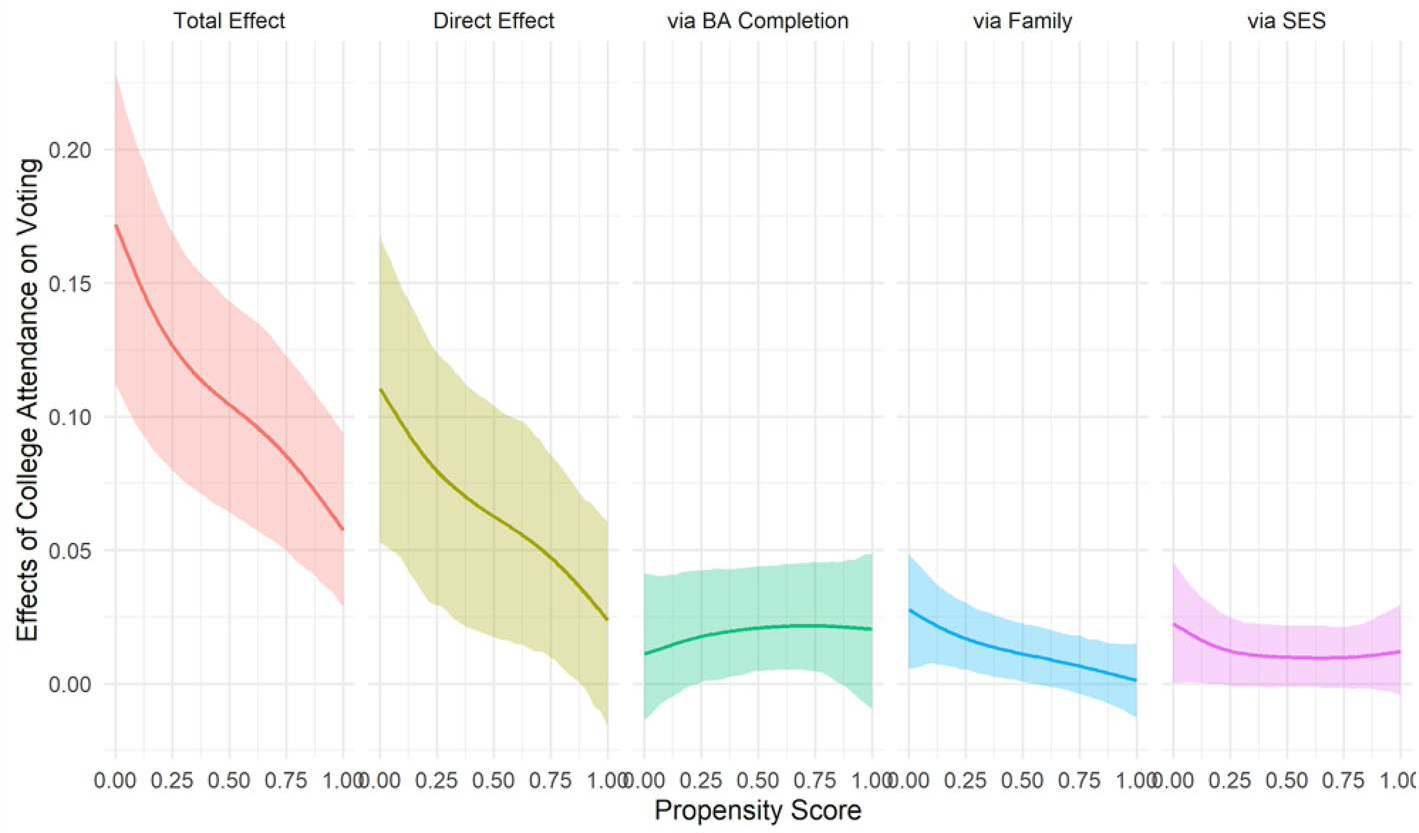
Heterogeneous total, direct, and indirect effects of college attendance on voting by propensity scores with 95% bootstrap confidence intervals (1000 iterations), NLSY79

We next assess heterogeneity by the propensity for 4-year college attendance in the effect of college attendance on self-reported voting. Larger effects of college on voting for those with higher propensity scores would suggest that greater increases in self-reported voting accrue to those more likely to attend college; larger effects for those with lower propensity scores, on the other hand, would suggest that those unlikely to attend college gain the most. We present our results for the total, direct, and indirect effects of college attendance in Figs. 3 and 4. In the left panel of Fig. 3, the dotted and dashed lines show that the probability of voting increases with the propensity of attending college regardless of attendance status. However, the probability of voting is particularly low for high-school graduates with a low propensity to attend college. As a result, the effect of college attendance on voting is higher among low-propensity than among high-propensity individuals, for whom the probability of voting is relatively high whether or not they attended college.^6^ The right panel depicts how the effect of college attendance, estimated as the difference between the estimated probability of self-reported voting with and without college attendance, varies by the propensity score. The total estimated effect of attendance on voting for respondents with the lowest propensity to attend is about 17 percentage points and decreases to about 6 percentage points for those with the highest propensity scores.

Figure 4 shows that the direct effect of college attendance on voting follows a similar pattern. Individuals who are least likely to attend college experience larger direct effects of college attendance on voting (about 12 percentage points) than those who are most likely to attend college (about 3 percentage points). We do not, however, find much heterogeneity in the indirect effects via degree completion, family, or SES. Thus, the strong pattern of negative selection in the total effect of college attendance on voting is primarily due to negative selection in the direct effect of college. The effect of college attendance on voting appears to operate largely through pathways other than degree completion, family formation, or socioeconomic attainment. These results are consistent with the hypothesis that college education increases political participation directly through socialization and the development of civic literacy during college, especially for individuals from socioeconomically disadvantaged backgrounds.

To assess the robustness of our estimates of heterogeneity in the total and path-specific effects by the propensity score, we also estimated heterogeneity in total, direct, and mediating effects by two key covariates that shape selection into college: parental income and measured cognitive ability. These results are presented in Online Appendix Fig. C.1 and C.2, respectively. Individuals with higher parental income or higher measured ability have lower total and direct effects of college attendance on voting than individuals with lower parental income or measured ability scores. Estimates of heterogeneity in the total, direct, and indirect effects of college attendance by parental income and cognitive ability align with the effects by the propensity of college.

### Supplementary Analyses on Heterogeneous Effects of College on Voting

**Fig. 5:**
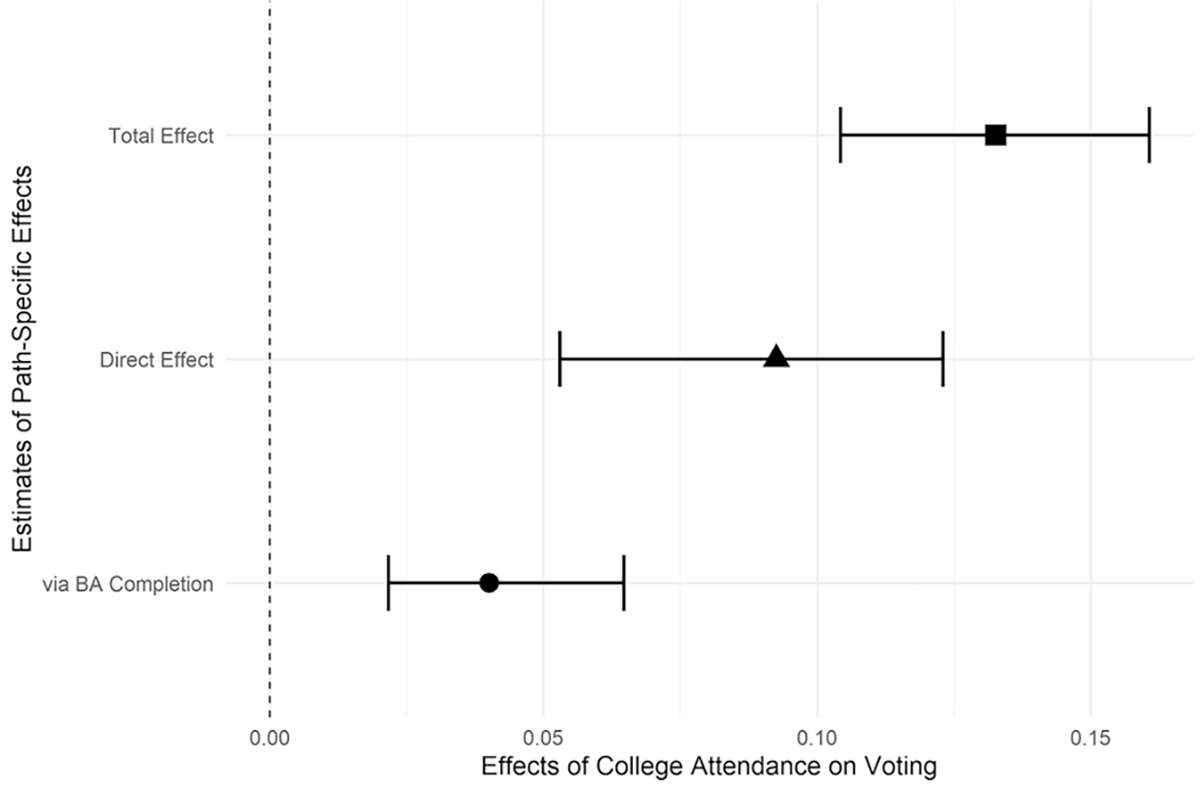
Estimated path-specific effects of college attendance on voting with 95% bootstrap confidence intervals (1000 iterations), NLSY97

**Fig. 6:**
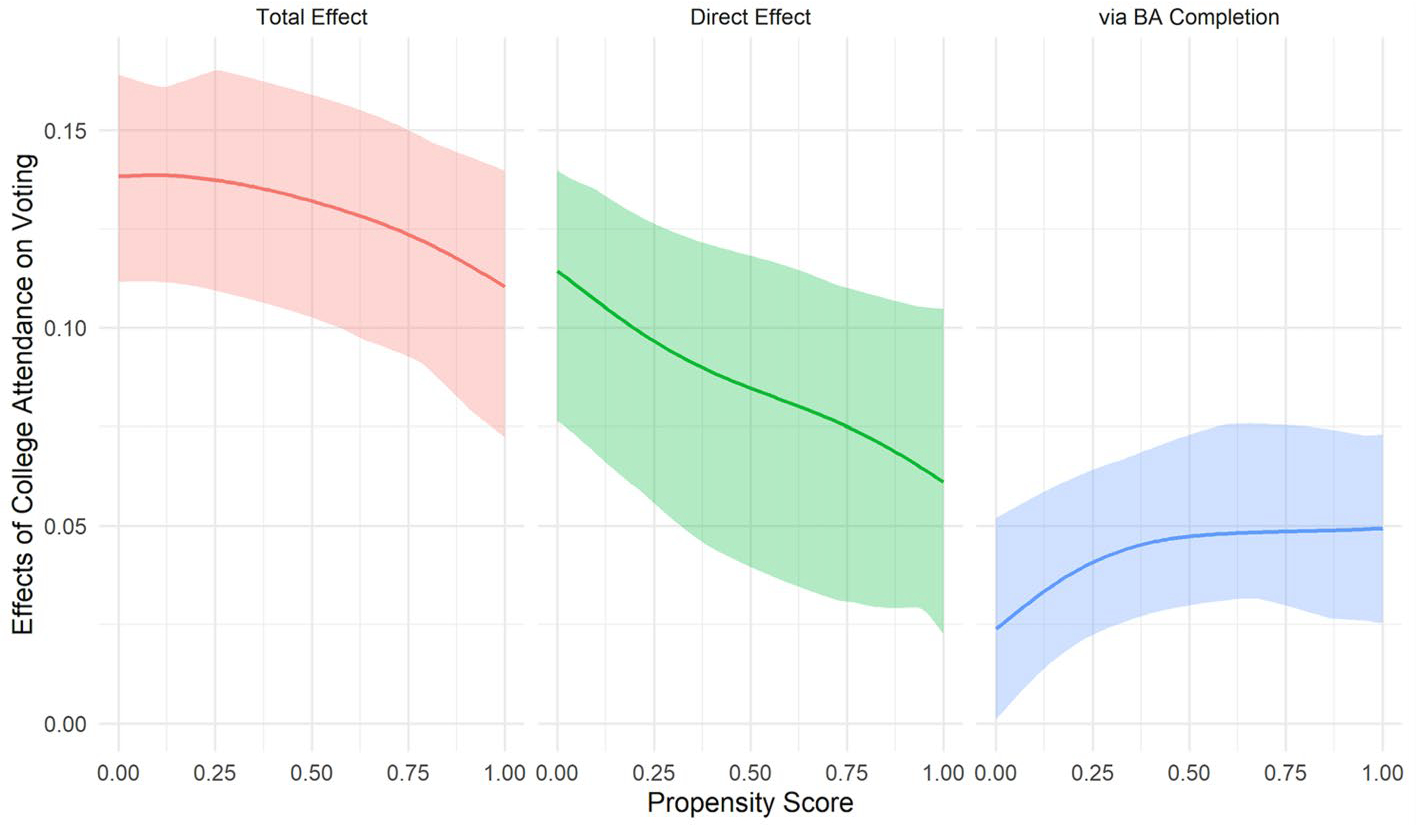
Heterogeneous total, direct, and indirect effects of college attendance on voting by propensity scores with 95% bootstrap confidence intervals (1000 iterations), NLSY97

The NLSY79 respondents were born in the early 1960s. While this gives us sufficient time to assess the mediating effects of post-college experiences on political participation, the experiences of this cohort may not well characterize those of more recent cohorts. Thus, we conduct a parallel analysis using the NLSY97 cohort, who were born in the 1980s, and who were asked about their participation in the 2010 general election. As noted earlier, the timing of the 2010 election does not allow us to assess the mediating effects of family and SES for this cohort. We thus restrict this supplementary analysis to assessing heterogeneity in the total effect of college attendance and the mediating effect via college completion. We show the results of the total and mediating effects in Figs. 5 and 6. Overall self-reported voting rates are lower in this sample (see Online appendix Table A.1), presumably because the NLSY97 respondents were much younger (i.e., in their late 20 s to early 30 s) in 2010 than the NLSY79 respondents were in 2006 (i.e., in their early 40 s), and voter turnout tends to increase with age (U.S. Bureau of the Census, 2020a). Still, results from these analyses largely align with results from the NLSY79 cohort. The total effect of attendance on voting (see Fig. 5) is about 13 percentage points, and the direct effect comprises about two-thirds of that effect (9 percentage points). Our results on effect heterogeneity for the NLSY97 cohort (see Fig. 6) are consistent with those reported in Figs. 3 and 4. The total and direct effects of college attendance, but not the mediating effect via bachelor’s degree completion, decrease as the propensity of college attendance increases.

### Sensitivity Analyses

We conducted two sets of sensitivity analyses to address potential biases in our results. First, to address the impact of voter overrepresentation in our sample, we replicated our main NLSY79 and NLSY97 analyses with an imputed measure of voting. Rather than omitting individuals who did not answer the voting item, we impute all individuals who were missing data for the voting question (either due to missing in the wave or missing the specific item) as non-voters. This assumption is grounded in previous research showing that individuals who do not participate in surveys tend to vote at lower rates than those who participate (Goldberg & Sciarini, 2019; Lahtinen et al., 2019). Still, this is a very strong assumption and is likely to overrepresent non-voters.

As with our main analyses, these results suggest a pattern of negative selection in the direct effects of 4-year college attendance on voting for both cohorts. However, they indicate that if the NLSY respondents on the voting item were much more likely to vote than those who were missing on that item, our results might have underestimated the mediating effects of college completion and socioeconomic attainment while overestimating the direct effects of college attendance. These auxiliary analyses do not address the issue of potential overrepresentation of voters in the initial survey, as they are conducted on a sample that remained in the survey long enough to provide information on college-going. Nevertheless, they help us understand the potential consequences of voter overrepresentation in the follow-up surveys. Even assuming this very high level of voter overrepresentation bias, our primary finding that low-propensity college-goers experience a greater increase in voting due to a larger direct effect of college attendance remains robust. See Online Appendix D for the full results of these analyses.

Second, our causal mediation analyses rest on the strong and untestable assumption that no unobserved confounding exists for any of the causal relationships represented in Fig. 1. Although we have adjusted for an array of baseline covariates in our analyses, there may still be unobserved individual attributes that affect both college attendance and political participation, or that affect both the mediators and political participation. We therefore conducted sensitivity analyses that explore the degree to which our estimates of total and direct effects are robust to the presence of unobserved confounding of the treatment-outcome and mediator-outcome relationships. Specifically, we use the bias factor approach developed by VanderWeele (2010) and VanderWeele & Arah (2011) (see Brand et al. (2019) for a recent application).

We begin by assessing the impact of an unobserved confounder of the treatment-outcome relationship. We show that if an unobserved trait increases voting by 10 percentage points and differs across college attendance status by 20 percentage points, the bias-adjusted estimate of the total effect will still be statistically significant and substantively similar to the original estimate. Next, we assess the impact of bias due to an unobserved confounder of the mediator-outcome relationship. For example, neighborhood-level social capital, which is not observed in this study, may affect both the mediators and political participation. Results from this analysis suggest that our main conclusion of a substantial direct effect of college attendance on voting is fairly robust to such potential confounding. We further show that, given large propensity score differences in the effect of college attendance on voting (as high as 11 percentage points), our finding of negative selection in the total and direct effects of college attendance is unlikely to be explained away by differential selection bias. Additional details and full results of these analyses are presented in Online Appendix D.

## Discussion and Conclusion

In the US, more-educated and higher-SES citizens persistently vote more than less-educated and lower-SES citizens (Leighley & Nagler, 2013). In this study, we asked whether 4-year college attendance might reduce inequality in voter turnout by increasing voting rates among those from disadvantaged backgrounds and how post-college socioeconomic attainment contributes to this process. Using a machine-learning-assisted causal mediation analysis, we examined how bachelor’s degree completion, family formation and stability, and various measures of socioeconomic attainment mediate the effect of college on voting, and the extent to which the total, direct, and indirect effects of college vary across individuals with different likelihoods of college attendance.

Our analyses of the NLSY79 cohort yield three notable findings. First, the estimated direct effect of college on self-reported voting is large, constituting about two-thirds of the total effect of attendance. If our models fully account for selection into college and selection into the mediators, this finding suggests that the college experience directly enhances voter turnout. Second, degree completion, post-college family characteristics, and socioeconomic attainment mediate the effect of college on self-reported voting, but the impact is smaller than the direct effect. Third, the total effect of college on voting exhibits a strong pattern of negative selection. Individuals with a lower likelihood of attending college, who tend to have more disadvantaged backgrounds, experience greater increases in self-reported voting due to college attendance than individuals from more privileged backgrounds. We find that the negative selection in the total effect is primarily due to a strong direct effect of college among low-propensity individuals. Our results suggest that self-reported voting increases most among individuals who are least likely to attend college not because they have the largest socioeconomic returns, but because they benefit more from the opportunities provided and skills developed during college than their more advantaged peers.

With a rich set of pre- and post-college variables and its longitudinal structure, the NLSY79 enables us to assess the long-term effects of college and how they unfold over the life course. Nevertheless, the NLSY79 cohort attended college during the 1980s, potentially reducing the direct relevance of our results to more current effects of higher education. To address this challenge, we ran supplementary analyses on the more recent NLSY97 cohort, who attended college in the early 2000s. Due to the young age of the NLSY97 survey respondents, we cannot examine the mediating pathways via family and socioeconomic attainment. Still, these results support our finding of negative selection in the direct effect of college attendance on voting and provide evidence that this effect appears relatively early in adulthood.

Taken together, our findings suggest that the expansion of higher education may reduce inequality in voter turnout, even when individuals do not complete a 4-year degree or attain high socioeconomic status. Despite extensive speculation on the processes through which college affects voting, prior work has not assessed the mediating role of post-college socioeconomic attainment. Thus, our study makes important inroads into identifying the potential civic benefits of expanding higher education to relatively disadvantaged groups and the conditions upon which those benefits depend. Scholars should continue to examine the mediating role of socioeconomic attainment in college’s additional civic consequences, such as other forms of political participation, volunteering, and pro-social attitudes, and how the direct and mediating effects vary across the population.

At least three potential sources of bias may have affected our results. First, some studies have suggested that vote overreporting among more educated respondents biases the estimates of college effects on voting (Ansolabehere & Hersh, 2012; Enamorado & Imai, 2019). However, more recent studies using high-quality data have found that the survey turnout gap is driven more by the overrepresentation of voters than by overreporting (Goldberg & Sciarini, 2019; Lahtinen et al., 2019). This recent work suggests that upward bias in our estimates of college attendance on self-reported voting is likely limited. Moreover, our focus is on heterogeneity in the effect of college. Previous research has not provided evidence for greater bias among low-propensity compared to high-propensity college-goers. Without data on the actual voting behavior of respondents and non-respondents, we cannot ascertain the level of bias in our estimates. Sensitivity analyses suggest that if there was a significant overrepresentation of voters in the sample who responded to our outcome variables, our results might have overstated the total and direct effects of college attendance on voting. However, the finding of negative selection in the direct effect remains robust.

Second, our analyses rest on the strong and untestable ignorability assumption, i.e., that no unobserved confounding exists for the total, direct, and indirect effects of college on voting. Because we cannot expect to observe and adjust for all possible confounders of the total and mediating effects of college on political participation, we ran a series of sensitivity analyses to understand how our estimates would respond to unmeasured factors (see Online Appendix D). The results suggest minimal unobserved confounding. We show that failing to adjust for an unobserved confounder in the treatment-outcome relationship or the mediator-outcome relationship is unlikely to meaningfully impact our estimates. Third, our estimates of mediating effects may be underestimated due to measurement error of family characteristics or socioeconomic attainment. Such measurement error would result in an upward bias in the direct effect of college on voting (Vanderweele et al., 2012). However, our analyses include multiple measures for each of our mediators and use adaptive machine learning models, thus reducing the likelihood that our results suffer from substantial bias due to measurement error.

While our analyses provide strong support for a direct effect of 4-year college on voting, we cannot adjudicate between specific explanations for this effect. Some have suggested that this direct effect stems from the college experience, including socialization on campus (Bowman, 2011; Chatfield & Henderson, 2016; Glynn et al., 2009), the development of civic and political literacy during college (Cassel & Lo, 1997; Galston, 2001; Hillygus, 2005), or pressure to conform to their more politically active social networks’ norms and expectations (Hansen & Tyner, 2019; Kingston et al., 2003; Rolfe, 2012). Given that we find stronger direct effects for lower-propensity college-goers, future studies in this area would benefit from assessing which direct processes are particularly salient for this population. For example, the change in social pressure to vote may be largest for relatively disadvantaged college-goers, or the increase in voting may reflect a response to social interactions that incite activism (Baker & Blissett, 2018). Although we cannot distinguish these explanations, our results suggest that the direct effects of college on voting, and ostensibly the social norms and networks that maintain them, are conceptually distinct from various measures of family formation and socioeconomic attainment.

While we examine heterogeneity by individual characteristics, future studies may additionally examine the role of different college experiences. For example, there is some evidence that online coursework is associated with reduced civic engagement relative to inperson instruction (Chatfield & Henderson, 2016). If limited campus time reduces the civic impact of college, the effects of college on voting may be similarly smaller for commuter students or those who work full time due to less exposure to campus experiences. Since relatively disadvantaged college-goers are more likely to commute (Schudde, 2011), an examination of commuter students may provide additional insight into differential effects within subpopulations of low-propensity college-goers as well as the mechanisms that lead to civic engagement. Finally, while we focus on 4-year college attendance and bachelor’s degree completion, recent research has suggested that increased proximity to a community college leads to increased voting (Doyle & Skinner, 2017). Future research in this area may investigate whether the effects identified in the present study extend to alternative post-secondary pathways and degrees.

In the early years of American higher education, colleges were charged with developing an educated citizenry to maintain a vibrant democracy. Indeed, producing informed and active citizens has long been an expressed mandate of the US educational system. Still, much social science research, and recent rhetoric on college benefits, focuses on economic rather than civic returns to college. We find that college attendance increases self-reported voting and that most of that effect does not operate through degree completion or college-induced increases in socioeconomic status. In other words, civic returns to college do not hinge on its socioeconomic returns; instead, they appear to stem primarily from the college experience itself. Moreover, college attendance has a larger direct effect on voting for individuals who are less likely to attend college, i.e., those from relatively disadvantaged backgrounds. Our findings thus highlight that an exclusive focus on the economic benefits of college can mask the broader societal benefits of expanding higher education to disadvantaged youth.

## Supplementary Material

Supplementary material

## Figures and Tables

**Table 1:** Descriptive statistics of outcome, covariates, and mediators by college attendance, NLSY79

	Full analytic sample		Non-college attenders		College attenders	
	Mean	SD	Mean	SD	Mean	SD

*Outcome*						
Voted in 2006 election (binary 0/1)	0.69	-	0.61	-	0.84	-
*Pre-college covariates*						
Male (binary 0/1)	0.50	-	0.50	-	0.48	-
Black (binary 0/1)	0.14	-	0.16	-	0.11	-
Hispanic (binary 0/1)	0.05	-	0.06	-	0.04	-
Father’s education (0–20)	12.24	3.26	11.33	2.94	13.73	3.21
Mother’s education (0–20)	11.98	2.40	11.34	2.15	13.04	2.41
Don’t know father’s education (binary 0/1)	0.04	-	0.06	-	0.02	-
Father white collar occupation (binary 0/1)	0.27	-	0.17	-	0.44	-
Parental income 1979, divided by 100 (0–750)	220.22	127.63	192.68	110.07	265.22	140.87
Lived with both parents at 14 (binary 0/1)	0.76	-	0.73	-	0.82	-
Number of siblings (0–15)	3.02	2.06	3.24	2.20	2.66	1.76
Catholic background (binary 0/1)	0.32	-	0.32	-	0.34	-
Jewish background (binary 0/1)	0.01	-	0.01	-	0.02	-
Southern residence (binary 0/1)	0.32	-	0.32	-	0.32	-
Rural residence (binary 0/1)	0.23	-	0.25	-	0.20	-
ASVAB ability score (−2.5–2.4)	0.13	0.68	−0.11	0.62	0.53	0.56
High school college prep program (binary 0/1)	0.35	-	0.21	-	0.59	-
College aspirations (binary 0/1)	0.58	-	0.41	-	0.85	-
College expectations (binary 0/1)	0.46	-	0.27	-	0.78	-
Closest friend aspires to college (binary 0/1)	0.48	-	0.34	-	0.70	
Rotter locus of control (4–16)^a^	8.78	2.27	9.05	2.22	8.34	2.28
Rosenberg self-esteem (8–30)	22.22	3.84	21.74	3.70	22.95	3.95
High on delinquency scale (binary 0/1)	0.79	-	0.82	-	0.74	-
School disadvantage (0–99)	18.51	16.13	21.02	16.91	14.43	13.82
Percent students black/hispanic (0–100)	19.45	22.62	21.46	23.79	16.17	20.15
High on scale of traditional family values (binary 0/1)	0.21	-	0.23	-	0.16	-
Married by age 18 (binary 0/1)	0.02	-	0.03	-	0.00	-
Parent by age 18 (binary 0/1)	0.02	-	0.02	-	0.00	-
*Family formation mediators*						
Prop of time married 1990–1998 (0–1)	0.58	0.41	0.56	0.42	0.61	0.41
Prop of time unmarried parent 1990–1998 (0–1)	0.16	0.31	0.23	0.35	0.06	0.19
Number of family transitions 1990–1998 (0–14)	0.80	1.72	1.06	1.95	0.37	1.15
*Socioeconomic status mediators*						
Log of mean income, Age 35–40 (2.0–12.0)	10.19	0.89	9.98	0.87	10.51	0.85
Prop of time out of labor force 1998–2006 (0–0.2)	0.03	0.04	0.03	0.04	0.02	0.04
Prop of time in poverty 1998–2006 (binary 0/1)	0.08	0.21	0.11	0.24	0.03	0.10
Cum welfare received 1998–2006 (2014 $) (0–150,000)	2170	10,955	3141	13,266	586.88	4968
Count of jobs held 1990–2006 (1–40)	4.44	3.36	4.61	3.44	4.16	3.22
Job tenure at age 40 (0–1446)	322	314	311	317	340	310
Count of moved addresses 2000–2006 (0–16)	1.12	1.61	1.23	1.70	0.96	1.43
No home ownership 2004 (binary 0/1)	0.26	-	0.32	-	0.17	-
N	2961		1950		1011	

All descriptive statistics are weighted with the NLSY79 sample weight. Unless noted as binary, all variables are continuous

Dashes are used in place of standard deviations for binary variables

a Rotter locus of control is scaled so that higher values indicate less control

## Footnotes

1 The NLSY voting measure benefts from four factors. First, misreporting may be lower in reporting turnout for midterm than for presidential elections. Second, as noted above, misreporting may be lower in nonpolitical surveys like the NLSY relative to political surveys like the American National Election Studies. Third, accuracy is greater in face-to-face interviews, like the NLSY, than in phone interviews (DeBell et al., 2020). Fourth, the NLSY question wording is intended to reduce social desirability pressures by allowing nonvoters to express their identity as habitual voters (option 2) and to indicate that they voted only if they were sure.

2 If we code all those who did not respond as non-voters, 53 percent report voting. This estimate is slightly below that of Current Population Survey (CPS) for the same education and age groups (i.e., 57 percent, authors’ calculations). The CPS voting measure also codes missing as non-voters. However, the CPS is a cross-sectional survey and thus those missing are not missing due to sample attrition, as is true when we code survey non-response as non-voters in the NLSY. The voting gap between college and non-college graduates is roughly the same (i.e., about 20 percentage points). Thus, even if the sample is under-representing non-voters, within-sample analyses may still be reasonable if we believe that the CPS is less susceptible to voter overrepresentation and overreporting.

3 Examining traditional-aged college students allows sufcient time for respondents to realize their post-college socioeconomic status and family formation. However, we acknowledge that this restriction misses some non-traditional aged college students who are more socioeconomically disadvantaged and less likely to vote. To examine whether these college-goers experience similar efects of college on voting, we ran additional analyses examining the efect of college attendance by age 25, and the mediating efect of bachelor’s degree completion by age 30. Results from this analysis are nearly identical to our main analyses.

4 This notation (with *CY*, *LY* adjacent, and with an arrow from M → Y) indicates that the paths from *C* to *Y* and from *L* to *Y* encompass both the direct pathway from the mediator to the outcome, as well as indirect paths through subsequent mediators. The path from *M* → *Y* includes only the direct pathway from the last mediator to the outcome.

5 We acknowledge the possibility that enrollment in a two-year college also increases voting. To examine whether analyses are sensitive to expanding our treatment to include two-year attendance, we estimated the effect of 2- or 4-year attendance by age 25, and the mediating effect of bachelor’s degree completion by age 30. The estimated total effect from this analysis is somewhat smaller, and the estimated direct effect is about half of the total effect. This suggests that the effect of 2-year college attendance on voting are smaller than the effect of 4-year college attendance.

6 Analyses that estimate the effect of any 2- or 4-year college attendance on voting produce a similar pattern of heterogeneity.
