# Supplementary material for: How, and For Whom, Does Higher Education Increase Voting?
Source: Res High Educ. Author manuscript; Available in PMC 2024 Sep 12. (PMC11392036; doi:10.1007/s11162-022-09717-4)
Supplement: Supplementary material [file NIHMS1948903-supplement-Supplementary_material.docx]

## Appendix A: NLSY97 Cohort Descriptive Statistics

Table A.1: Descriptive Statistics of Outcome, Covariates, and Mediators by College Attendance, NSLY97

|  |  | Full Analytic Sample | | Non-College Attenders | | College Attenders | |
| --- | --- | --- | --- | --- | --- | --- | --- |
|  |  | Mean | SD | Mean | SD | Mean | SD |
| Outcome | |  |  |  |  |  |  |
|  | Voted in 2010 election (binary 0/1) | 0.40 | - | 0.31 | - | 0.54 | - |
| Pre-College Covariates | |  |  |  |  |  |  |
|  | Male (binary 0/1) | 0.51 | - | 0.54 | - | 0.45 | - |
|  | Black (binary 0/1) | 0.16 | - | 0.19 | - | 0.12 | - |
|  | Hispanic (binary 0/1) | 0.13 | - | 0.17 | - | 0.08 | - |
|  | Father's Education (0-20) | 12.81 | 3.03 | 11.94 | 2.71 | 14.28 | 2.98 |
|  | Mother's Education (0-20) | 12.83 | 2.74 | 12.07 | 2.49 | 14.11 | 2.66 |
|  | Parental Income 1997 (0-246000) | 50597 | 40387 | 39764 | 31251 | 68768 | 46925 |
|  | Lived with Both Parents at 14 (binary 0/1) | 0.52 | - | 0.42 | - | 0.69 | - |
|  | Number of Siblings (0-45) | 2.54 | 2.13 | 2.81 | 2.31 | 2.09 | 1.7 |
|  | Catholic Background (binary 0/1) | 0.26 | - | 0.25 | - | 0.27 | - |
|  | Jewish Background (binary 0/1) | 0.01 | - | 0.01 | - | 0.03 | - |
|  | Southern Residence (binary 0/1) | 0.34 | - | 0.35 | - | 0.31 | - |
|  | Rural Residence (binary 0/1) | 0.21 | - | 0.20 | - | 0.21 | - |
|  | ASVAB Ability Score (-3.2-2.6) | -0.20 | 0.65 | -0.40 | 0.60 | 0.14 | 0.60 |
|  | Self-Reported High School GPA (0-4) | 2.84 | 0.82 | 2.54 | 0.79 | 3.34 | 0.60 |
|  | High School College Prep Program (binary 0/1) | 0.32 | - | 0.18 | - | 0.56 | - |
|  | Believes School is Safe | 0.33 | - | 0.27 | - | 0.44 | - |
|  | Believes Teachers are Good (binary 0/1) | 0.18 | - | 0.15 | - | 0.24 | - |
|  | Friend's College Completion Aspirations (binary 0/1) | 0.22 | - | 0.18 | - | 0.30 | - |
|  | High on Delinquency Scale (binary 0/1) | 0.55 | - | 0.62 | - | 0.44 | - |
|  | Married by Age 18 (binary 0/1) | 0.02 | - | 0.03 | - | 0.00 | - |
|  | Parent by Age 18 (binary 0/1) | 0.07 | - | 0.10 | - | 0.01 | - |
|  | N | 7620 | | 5073 | | 2547 | |
| *Notes:* All descriptive statistics are weighted with the NLSY97 sample weight. Unless noted as binary, all variables are continuous. Dashes are used in place of standard deviations for binary variables. | | | | | | | |

## Appendix B: Identification of Path-Specific Effects

Let $A$ denote treatment (i.e., four-year college attendance), $Y$ the outcome of interest (i.e., voted in 2006), $X$ the vector of pretreatment covariates,$C$ the four-year college completion mediator, $L$ the set of mediators reflecting family formation and stability, and $M$ the set of mediators reflecting SES. The causal paths (a), (b), (c), (d) shown in Figure 1 can be represented as (a) $A\to Y$; (b) $A\to CY$; (c) $A\to LY$; and (d) $A\to M\to Y$. As shown in Zhou and Yamamoto (2022), under the assumption of no unobserved confounding, the corresponding path-specific effects can be identified as

$$\tau_{A\to Y}=E\left[ E\left[ E\left[ Y | X,A=1,C, L,M \right] | X,A=0 \right]-E\left[ Y | X,A=0 \right] \right], \left( B.1 \right)$$

$$\tau_{A\to M\to Y}=E\left[ E\left[ E\left[ Y | X,A=1,C,L \right] | X,A=0 \right]-E\left[ E\left[ Y | X,A=1,C,L,M \right] | X,A=0 \right] \right], (B.2)$$

$$\tau_{A\to LY}=E\left[ E\left[ E\left[ Y | X,A=1,C \right] | X,A=0 \right]-E\left[ E\left[ Y | X,A=1,C,L \right] | X,A=0 \right] \right], (B.3)$$

$$\tau_{A\to CY}=E\left[ E\left[ Y | X,A=1 \right]-E\left[ E\left[ Y | X,A=1,C \right] | X,A=0 \right] \right]. (B.4)$$

To gain some intuition of how the above formulas work, let us consider equation (B.1). Here, the inner expectation $E\left[ X,A=1,C, L,M \right]$ captures the predicted voting outcome given pretreatment covariates $X$, college completion status $C$, family characteristics $L$, and SES $M$ had everyone, contrary to fact, attended college. The outer expectation $E\left[ X,A=0 \right]$ is the conditional mean of this predicted value given pretreatment covariates $X$ among non-college goers. Thus, this term reflects a counterfactual of what might be expected for those who did not attend college had they done so, but with their mediators (college completion status, family characteristics, and SES) kept at their realized values under non-attendance ($A=0$). Then, by subtracting from this term the predicted voting outcome given pretreatment covariates $X$ among non-college goers, we obtain the direct effect of college given pretreatment covariates $X$, the mean of which (the outmost expectation) constitutes the average direct effect of college. Equations (B.2-B.4) can be understood analogously.

## Appendix C: Heterogeneity by parental Income and Ability


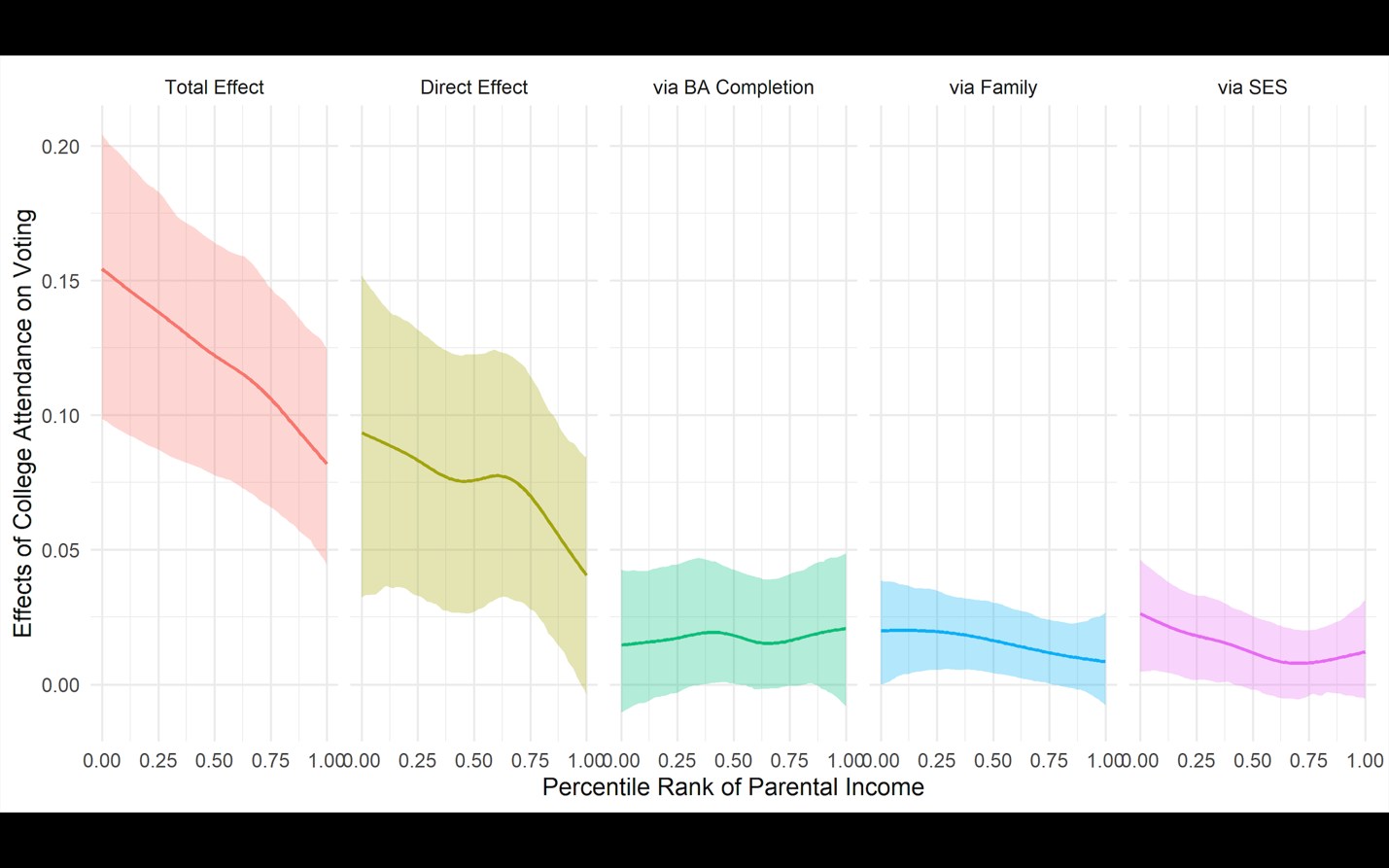


Figure C.1: Heterogeneous Total, Direct, and Indirect Effects of College Attendance on Voting by Parental Income with 95% Bootstrap Confidence Intervals (1,000 iterations), NLSY79


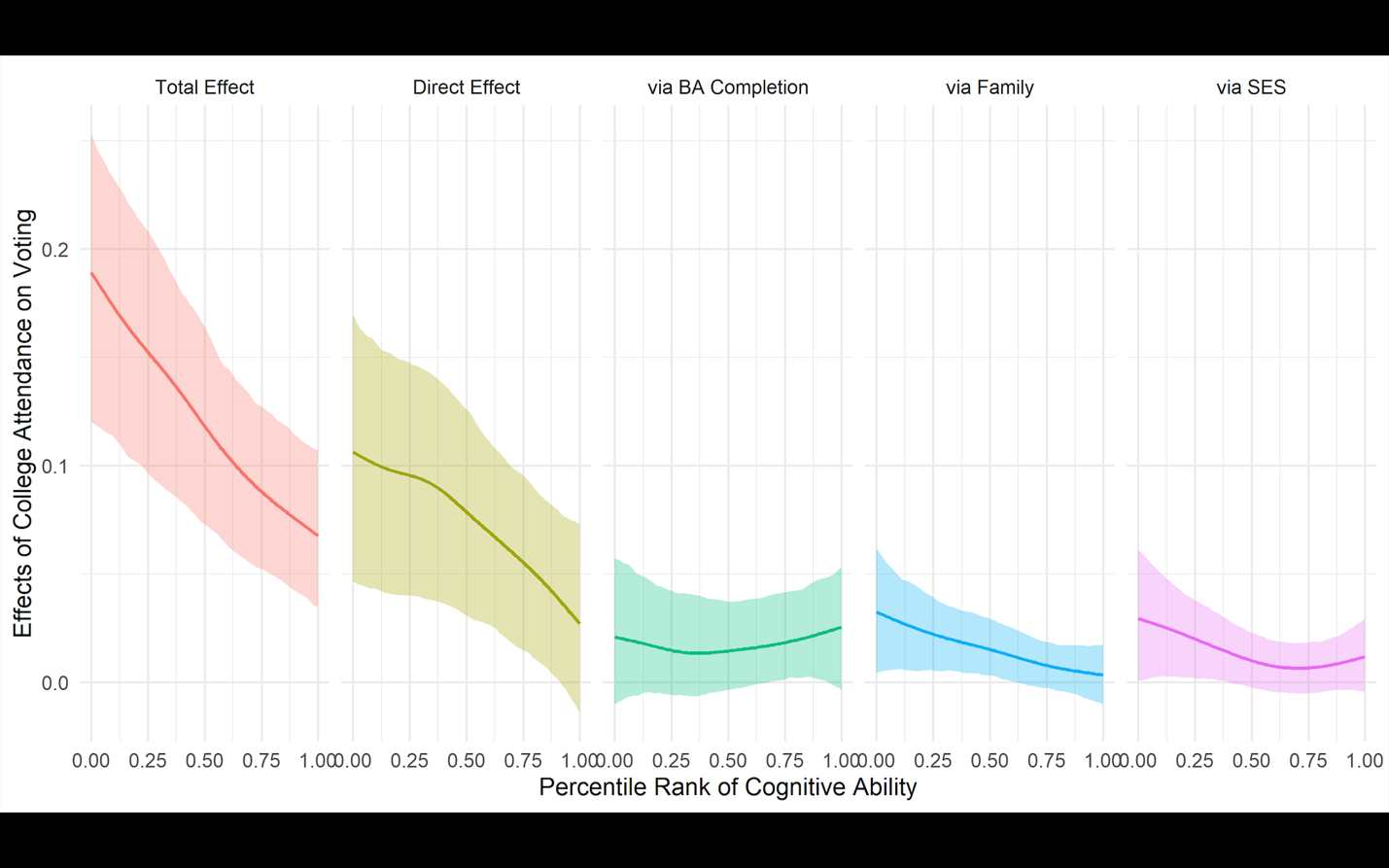


Figure C.2: Heterogeneous Total, Direct, and Indirect Effects of College Attendance on Voting by Cognitive Ability with 95% Bootstrap Confidence Intervals (1,000 iterations), NLSY79

## Appendix D: Results of Sensitivity Analyses

As we note in the main text, we replicated our main NLSY79 and NLSY97 analyses with an imputed measure of voting that addresses the potential impact of voter overrepresentation. Rather than omitting individuals who did not answer the voting item, we code all individuals who were missing data for the voting question (either due to missing in the wave or missing the specific item) as non-voters. This imputation assumption is grounded in previous research showing that individuals who do not participate in surveys tend to vote at lower rates than those who participate (Goldberg and Sciarini 2019; Lahtinen et al. 2019). Still, this is a very strong assumption and is likely to overrepresent non-voters.

We begin by replicating analyses for the NLSY79 sample. These results show some differences from the main results. First, the total effect of college on voting is about 8 percentage points (Figure D.1), compared to about 12 percentage points in the original sample. Second, the direct effect of college attendance on voting is smaller (about .03 compared to .08), and the mediating effect via college completion is larger (about .04 compared to .02) (Figure D.2). Finally, there remains a pattern of negative selection in the direct effect, but there is now a modest positive pattern in the mediating effects of college completion and SES. The corresponding results for the NLSY97 sample are shown in Appendix Figures D.3 and D.4. The overall estimated total and direct effects, and the mediating effect via college completion are all quite similar to the estimates from our main analyses, at around 13, 9, and 4 percentage points, respectively. However, the pattern of negative selection by the propensity score in the total effect is much less pronounced than in the non-missing sample (see Figure D.4). Yet, we still find a pattern of negative selection in the direct effect, and it appears that the lack of effect heterogeneity in the total effect is due to a positive slope in the mediating effect via college completion.

We also assess the potential bias due to unobserved confounding variables. First, we address potential bias in the treatment-outcome relationship. Although we have adjusted for an array of baseline covariates in our analyses, there may still be unobserved individual attributes that affect both college attendance and political participation. For simplicity, let us consider a binary unobserved confounder $U$, say some unmeasured personality trait. Under the simplifying assumption that the average effect of $U$ on voting does not depend on treatment status $A$, the bias for the total effect conditional on the baseline covariates $X=x$ is given by

$$B_{total}\left( x \right)=\gamma\delta$$

where

$$\gamma=Pr\left[ Y=1 | U=1,A,X=x \right]-Pr\left[ Y=1 | U=0,A,X=x \right]$$

and

$$\delta=Pr\left[ U=1 | A=1,X=x \right]-Pr\left[ U=1 | A=0,X=x \right].$$

That is, $\gamma$ is the difference in voting between individuals with and without unobserved characteristic $U$ conditional on treatment status $A$ and baseline covariates $X=x$, and $\delta$is the difference in the prevalence of $U$ between individuals with and without a college education conditional on baseline covariates $X=x$.

Given that a characteristic that facilitates college attendance is likely positively associated with voting, consider the case where $\gamma$ and $\delta$ are of the same sign. Here, the bias is positive, meaning the total effect of college attainment on voting is overestimated. Table D.1 shows the bias-adjusted estimates of the total effect across a range of plausible values for $\gamma$ and $\delta$. The estimated total effect of college attendance on voting is quite robust. Even if the unobserved trait $U$ increases voting by as much as 10 percentage points and the prevalence of $U$ differs by as much as 20 percentage points between college and non-college goers, the bias-adjusted estimate of the total effect will still be statistically significant and substantively similar to the original estimate.

Second, unobserved confounders may exist for the mediator-outcome relationship. For example, neighborhood-level social capital, an unobserved variable in this study, may affect both the mediators and political participation. In this case, while the total effect estimate may still be unbiased, the direct and indirect effects will likely be overestimated or underestimated. To explore the direction and magnitude of potential bias, we again use a bias factor approach (VanderWeele 2010). Here, we consider a binary unobserved confounder $U$, say social capital, for the mediator-outcome relationships. Under some simplifying assumptions, the bias for the direct effect conditional on the baseline covariates $X=x$ is given by

$$B_{direct}\left( x \right)=\gamma\delta$$

where

$$\gamma=Pr\left[ Y=1 | U=1,X=x,A,C,L,M \right]-Pr\left[ Y=1 | U=0,X=x,A,C,L, M \right]$$

and

$$\delta=Pr\left[ U=1 | A=1,X=x,C,L,M \right]-Pr\left[ U=1 | A=0,X=x,C,L,M \right].$$

Here, $\gamma$ is the difference in voting participation between individuals with and without social capital $U$ conditional on college status $A$, the baseline covariates $X=x$, and the mediators reflecting college completion (*C*), family formation and stability ($L$), and socioeconomic attainment ($M$), while $\delta$is the difference in the prevalence of $U$ between individuals with and without college attendance conditional on baseline covariates and the mediators.

Considering that social capital generally boosts political participation, let us assume, without loss of generality, that $\gamma>0$. The sign of $\delta$, however, may be either positive or negative. Social capital tends to be higher among individuals who attended college. Yet if both college attendance and social capital promote college completion, family formation and stability, and socioeconomic status, conditioning on these mediators may reduce or even reverse their association. Thus, we assess the bias term $B_{direct}(x)$ for both positive and negative values of $\delta$. Table D.2 shows the bias-adjusted estimates of the direct effect across a range of plausible values for $\gamma$ and $\delta$. The direct effect of college on voting will be underestimated if $\delta$ is negative and overestimated if $\delta$ is positive. Yet, our main conclusion that a substantial portion of the total effect of college attendance is direct is fairly robust. For example, if the unobserved confounder $U$ increases voting by 10 percentage points and the prevalence of $U$ differs by 10 percentage points between college and non-college goers with the same baseline characteristics and mediator values, the associated bias will only be 0.01, much smaller than the estimated direct effect.

The bias formulas presented above also inform the sensitivity of our results on treatment effect heterogeneity to unobserved selection. First, we note that if the $\gamma$ and $\delta$ parameters are constant across different values of the propensity score, then low- and high-propensity individuals will suffer the same amount of bias. In this case, our finding that the effect of college on voting is larger among low-propensity individuals will be unchanged. However, the $\gamma$ and $\delta$ parameters may differ between low- and high-propensity individuals. For example, the effect of an unmeasured characteristic on voting might be stronger among low-propensity individuals than among high-propensity individuals, for whom voting likely reflects a social norm and thus depends less on personality traits. If so, our finding of negative selection might be a result of differential selection bias. If we use $\gamma_{L}$ and $\delta_{L}$ to denote the $\gamma$ and $\delta$ parameters defined above for low-propensity individuals and $\gamma_{H}$ and $\delta_{H}$ for high-propensity individuals, the bias for the estimated difference in treatment effect between the two subpopulations will be

$$B_{diff}=\gamma_{L}\delta_{L}-\gamma_{H}\delta_{H}.$$

Our results indicated that the estimated total effect of college attendance decreases as the propensity score increases, ranging from .17 to .06. Thus, the difference in bias between low- and high-propensity individuals would have to be as large as .11 (.17-.06) to explain away effect heterogeneity. Given the range of plausible values for $\gamma_{L}\delta_{L}$ and $\gamma_{H}\delta_{H}$ (Table D.2), it is unlikely that unobserved confounding can explain away the degree of negative selection shown in Figure 3. The degree of negative selection in the estimated direct effect reported in Figure 4 likewise appears too large to be plausibly attributed to differential selection bias.


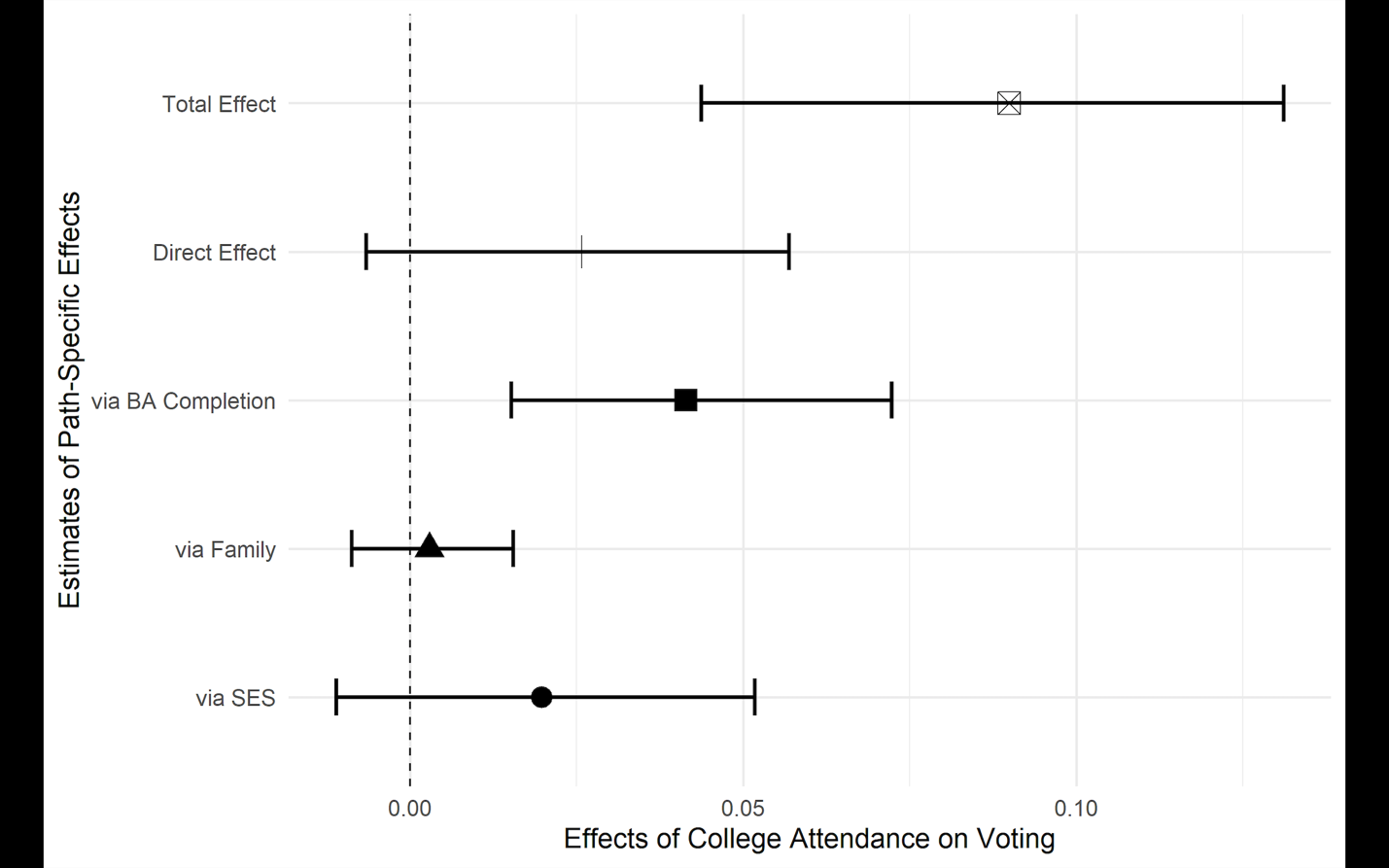


Figure D.1: Estimated Path-Specific Effects of College Attendance on Voting With 95% Bootstrap Confidence Intervals (1,000 iterations), NLSY79 Overrepresentation Bias Sample


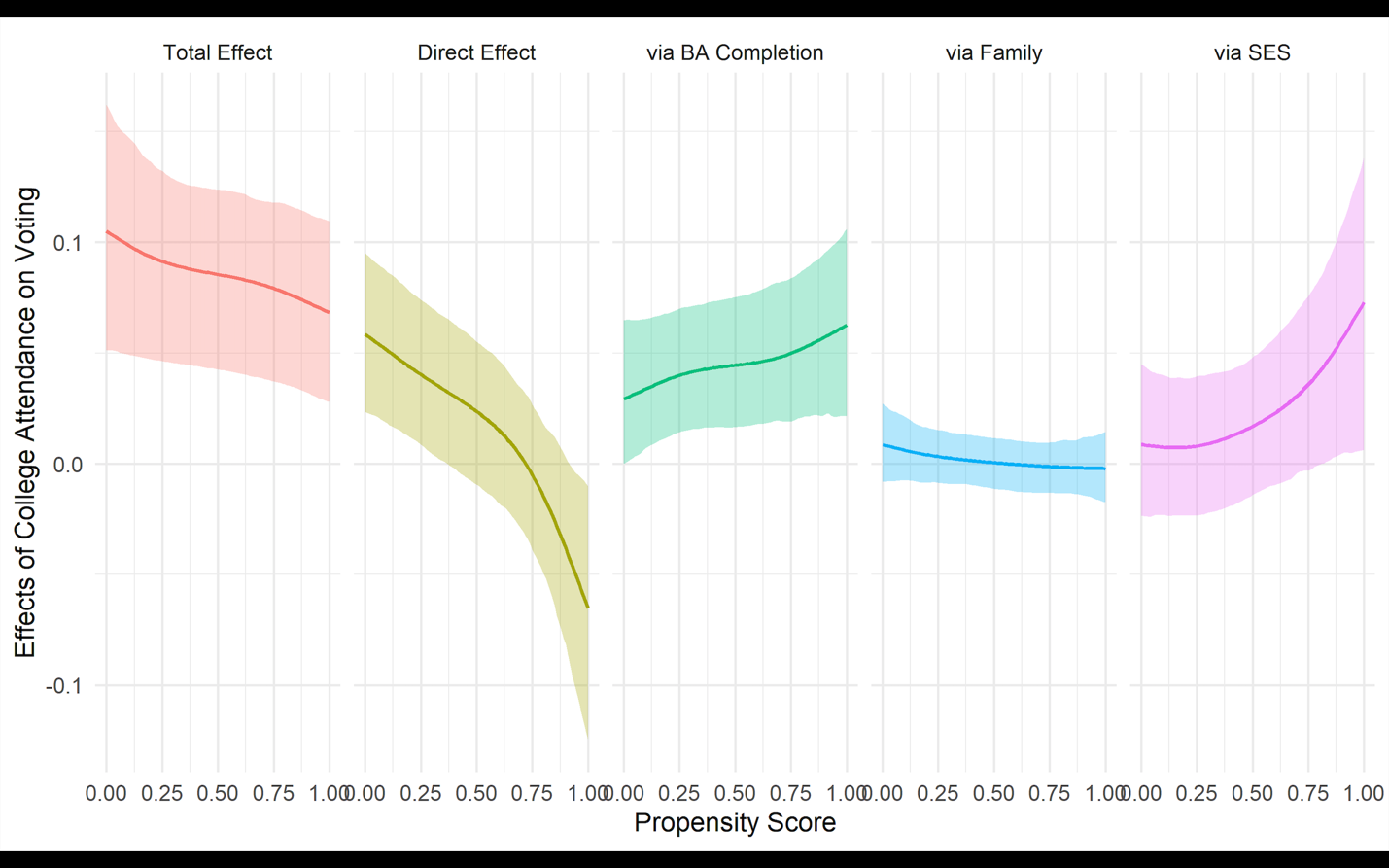


Figure D.2: Heterogeneous Total, Direct, and Indirect Effects of College Attendance on Voting by Propensity Scores with 95% Bootstrap Confidence Intervals (1,000 iterations), NLSY79

NLSY79 Overrepresentation Bias Sample


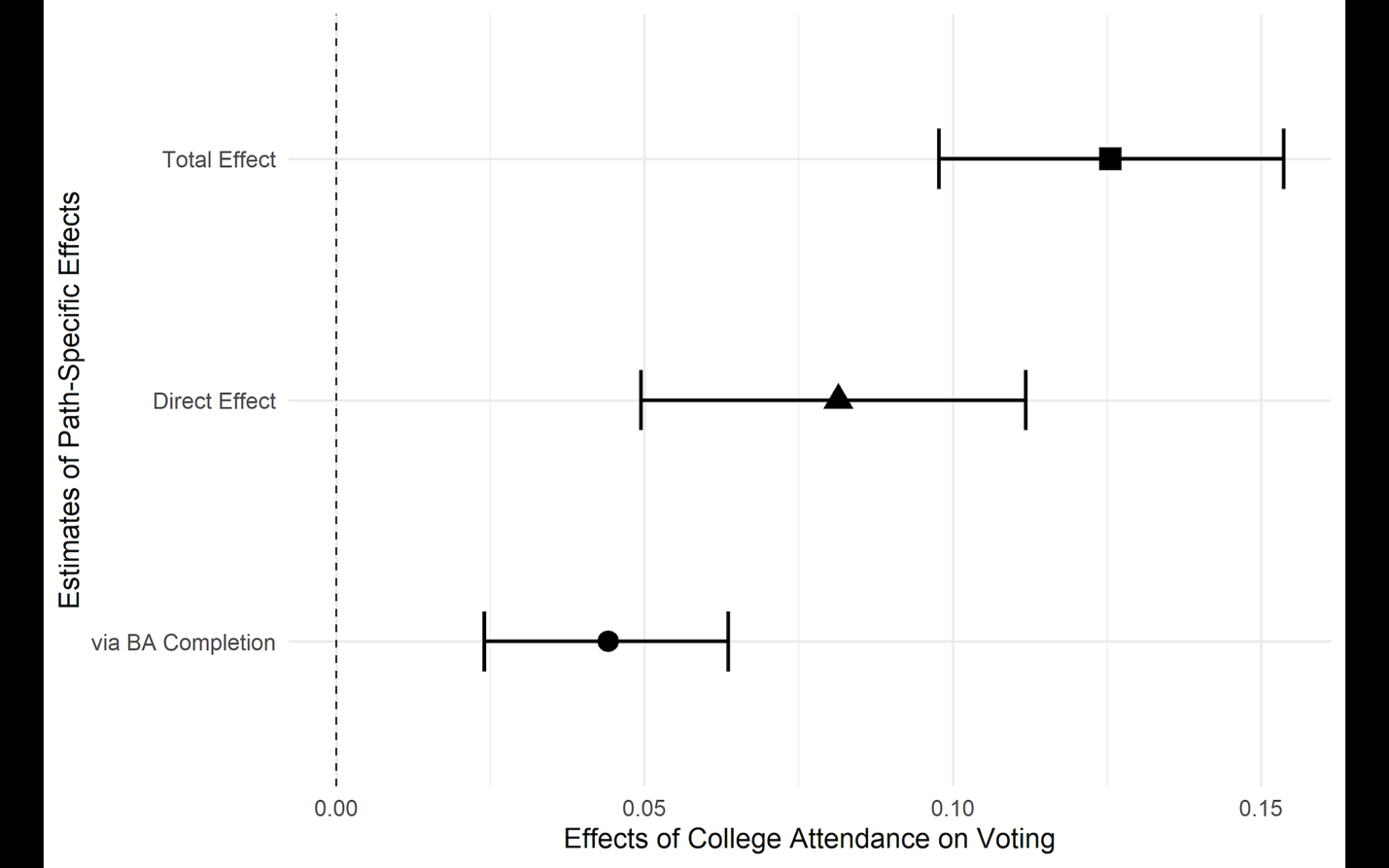


Figure D.3: Estimated Path-Specific Effects of College Attendance on Voting With 95% Bootstrap Confidence Intervals (1,000 iterations), NLSY97 Overrepresentation Bias Sample


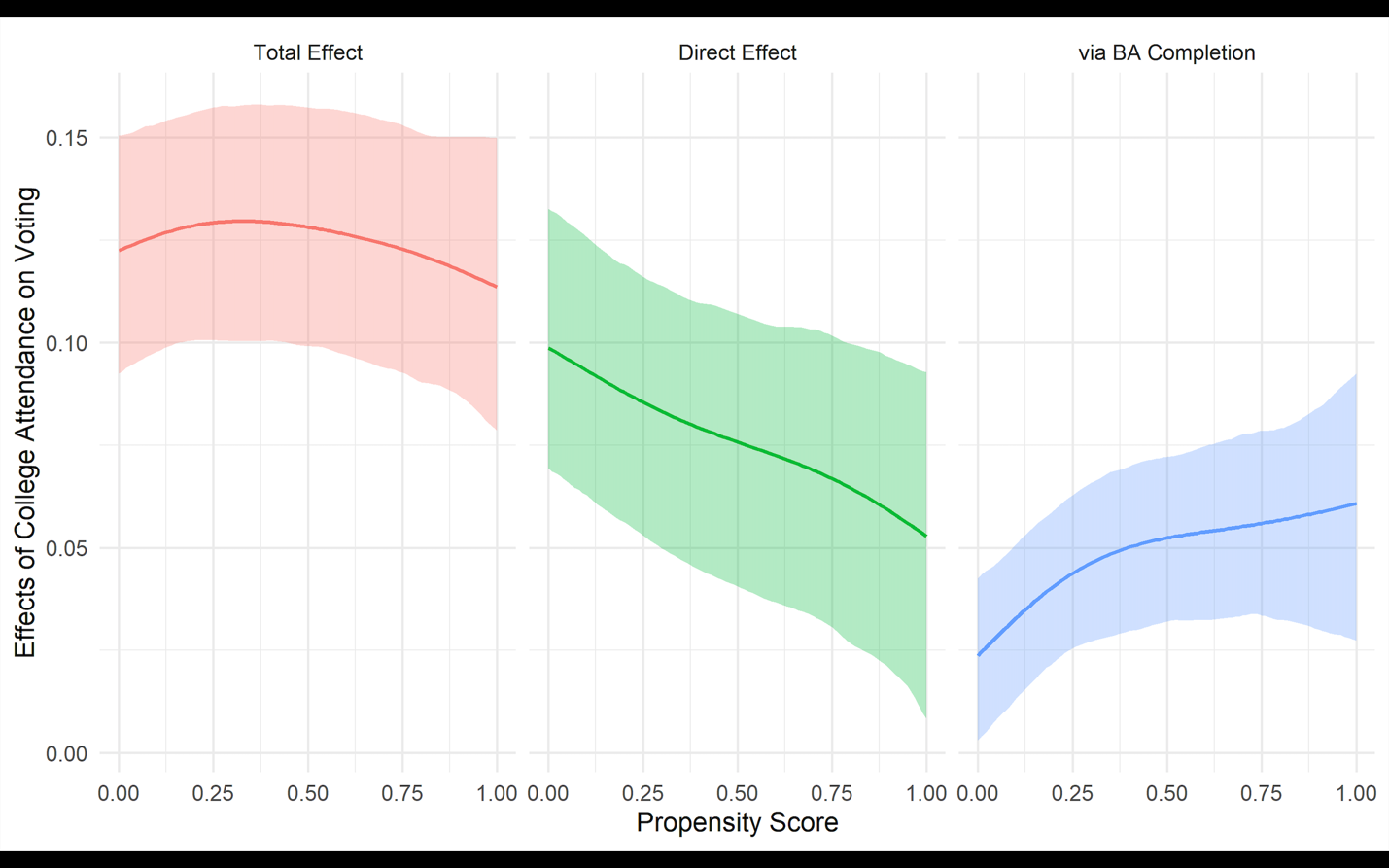


Figure D.4: Heterogeneous Total, Direct, and Indirect Effects of College Attendance on Voting by Propensity Scores with 95% Bootstrap Confidence Intervals (1,000 iterations), NLSY97 Overrepresentation Bias Sample

Table D.1: Sensitivity Results for Total Effects of College Attendance on Voting, NLSY79

| Sensitivity Parameters | | Bias | College Attendance | |
| --- | --- | --- | --- | --- |
| $\gamma$ | $\delta$ |  | Bias-adjusted Estimate | 95% Confidence Interval |
| 0.00 | 0.00 | 0.00 | 0.12 | (0.08, 0.16) |
| 0.05 | 0.05 | 0.00 | 0.12 | (0.07, 0.16) |
| 0.10 | 0.05 | 0.01 | 0.12 | (0.07, 0.16) |
| 0.20 | 0.05 | 0.01 | 0.11 | (0.07, 0.15) |
| 0.05 | 0.10 | 0.01 | 0.12 | (0.07, 0.16) |
| 0.10 | 0.10 | 0.01 | 0.11 | (0.07, 0.15) |
| 0.20 | 0.10 | 0.02 | 0.10 | (0.06, 0.14) |
| 0.05 | 0.20 | 0.01 | 0.11 | (0.07, 0.15) |
| 0.10 | 0.20 | 0.02 | 0.10 | (0.06, 0.14) |
| 0.20 | 0.20 | 0.04 | 0.08 | (0.04, 0.12) |

Table D.2: Sensitivity Results for Direct Effects of College Attendance on Voting, NLSY79

| Sensitivity Parameters | | Bias | College Attendance | |
| --- | --- | --- | --- | --- |
| $\gamma$ | $\delta$ |  | Bias-adjusted Estimate | 95% Confidence Interval |
| 0.00 | 0.00 | 0.00 | 0.07 | (0.03, 0.12) |
| 0.05 | -0.05 | 0.00 | 0.08 | (0.03, 0.12) |
| 0.10 | -0.05 | -0.01 | 0.08 | (0.03, 0.12) |
| 0.20 | -0.05 | -0.01 | 0.08 | (0.04, 0.13) |
| 0.05 | -0.10 | -0.01 | 0.08 | (0.03, 0.12) |
| 0.10 | -0.10 | -0.01 | 0.08 | (0.04, 0.13) |
| 0.20 | -0.10 | -0.02 | 0.09 | (0.05, 0.14) |
| 0.05 | 0.05 | 0.00 | 0.07 | (0.03, 0.11) |
| 0.10 | 0.05 | 0.01 | 0.07 | (0.02, 0.11) |
| 0.20 | 0.05 | 0.01 | 0.06 | (0.02, 0.11) |
| 0.05 | 0.10 | 0.01 | 0.07 | (0.02, 0.11) |
| 0.10 | 0.10 | 0.01 | 0.06 | (0.02, 0.11) |
| 0.20 | 0.10 | 0.02 | 0.05 | (0.01, 0.10) |
